# Genome-wide CRISPR screens for Shiga toxins and ricin reveal Golgi proteins critical for glycosylation

**DOI:** 10.1371/journal.pbio.2006951

**Published:** 2018-11-27

**Authors:** Songhai Tian, Khaja Muneeruddin, Mei Yuk Choi, Liang Tao, Robiul H. Bhuiyan, Yuhsuke Ohmi, Keiko Furukawa, Koichi Furukawa, Sebastian Boland, Scott A. Shaffer, Rosalyn M. Adam, Min Dong

**Affiliations:** 1 Department of Urology, Boston Children’s Hospital, Boston, Massachusetts, United States of America; 2 Department of Surgery, Harvard Medical School, Boston, Massachusetts, United States of America; 3 Department of Microbiology and Immunobiology, Harvard Medical School, Boston, Massachusetts, United States of America; 4 Department of Biochemistry and Molecular Pharmacology, University of Massachusetts Medical School, Worcester, Massachusetts, United States of America; 5 Mass Spectrometry Facility, University of Massachusetts Medical School, Shrewsbury, Massachusetts, United States of America; 6 Division of Genetics, Brigham and Women’s Hospital and Harvard Medical School, Boston, Massachusetts, United States of America; 7 Department of Biomedical Sciences, Chubu University College of Life and Health Sciences, Matsumoto, Kasugai, Aichi, Japan; 8 Department of Genetics and Complex Diseases, Harvard T.H. Chan School of Public Health, Boston, Massachusetts, United States of America

## Abstract

Glycosylation is a fundamental modification of proteins and membrane lipids. Toxins that utilize glycans as their receptors have served as powerful tools to identify key players in glycosylation processes. Here, we carried out Clustered Regularly Interspaced Short Palindromic Repeats (CRISPR)-Cas9–mediated genome-wide loss-of-function screens using two related bacterial toxins, Shiga-like toxins (Stxs) 1 and 2, which use a specific glycolipid, globotriaosylceramide (Gb3), as receptors, and the plant toxin ricin, which recognizes a broad range of glycans. The Stxs screens identified major glycosyltransferases (GTs) and transporters involved in Gb3 biosynthesis, while the ricin screen identified GTs and transporters involved in *N*-linked protein glycosylation and fucosylation. The screens also identified lysosomal-associated protein transmembrane 4 alpha (LAPTM4A), a poorly characterized four-pass membrane protein, as a factor specifically required for Stxs. Mass spectrometry analysis of glycolipids and their precursors demonstrates that LAPTM4A knockout (KO) cells lack Gb3 biosynthesis. This requirement of LAPTM4A for Gb3 synthesis is not shared by its homolog lysosomal-associated protein transmembrane 4 beta (LAPTM4B), and switching the domains between them determined that the second luminal domain of LAPTM4A is required, potentially acting as a specific “activator” for the GT that synthesizes Gb3. These screens also revealed two Golgi proteins, Transmembrane protein 165 (TMEM165) and Transmembrane 9 superfamily member 2 (TM9SF2), as shared factors required for both Stxs and ricin. TMEM165 KO and TM9SF2 KO cells both showed a reduction in not only Gb3 but also other glycosphingolipids, suggesting that they are required for maintaining proper levels of glycosylation in general in the Golgi. In addition, TM9SF2 KO cells also showed defective endosomal trafficking. These studies reveal key Golgi proteins critical for regulating glycosylation and glycolipid synthesis and provide novel therapeutic targets for blocking Stxs and ricin toxicity.

## Author summary

Shiga and Shiga-like toxins (Stxs) are a family of bacterial toxins and key virulence factors for the bacteria *Shigella dysenteriae* and enterohemorrhagic *Escherichia coli* (EHEC), which cause food poisoning throughout the world. Ricin is a plant toxin and a potential bioterrorism agent. Stxs recognize the host receptor glycolipid Gb3, while ricin utilizes a broad range of glycans as receptors. Here, we carried out genome-wide loss-of-function CRISPR-Cas9 screens using human cells to identify factors required for Stxs and ricin. Besides host factors previously known to be involved in the action of these toxins, our screens revealed three previously poorly characterized Golgi proteins: LAPTM4A, which is specifically required for Stxs, and TMEM165 and TM9SF2, which are required for both Stxs and ricin. Further characterization demonstrates that LAPTM4A is specifically required for biosynthesis of the glycolipid Gb3, potentially acting as an “activator” protein for the glycosyltransferase that synthesizes Gb3, whereas TMEM165 and TM9SF2 are likely required to maintain a proper environment within the Golgi for optimal activity of glycosyltransferases. These findings provide mechanistic insights to glycolipid biosynthesis and regulation of glycosylation levels in the Golgi and also reveal novel therapeutic targets for preventing Stxs and ricin intoxication.

## Introduction

The plant toxin ricin is derived from castor oil plant seeds. It has been utilized as a poison in criminal cases and is classified as a potential bioterrorism agent [1]. Shiga and Shiga-like toxins (Stxs) are a family of bacterial toxins including the prototype Stx, produced by the bacteria *Shigella dysenteriae*, and related Shiga-like toxins Stx1 and Stx2, produced by Shigatoxigenic strains such as enterohemorrhagic *E*. *coli* (EHEC) [2,3]. EHEC is a major pathogen responsible for food poisoning and causing abdominal cramps and bloody diarrhea, as well as the life-threatening complication of hemolytic uremic syndrome (HUS) [4].

Ricin and Stxs are structurally and evolutionarily distinct but share the same mode of action: both act as ribosomal RNA *N*-glycosidase and inhibit protein synthesis by cleaving the same adenine of 28S rRNA. Ricin is composed of an A chain (32 kDa) and a B chain (34 kDa), connected via a disulfide bond. The A chain is an *N*-glycosidase and the B chain is the receptor-binding domain. Stxs are A-B5 bacterial toxins [2,3], composed of an A chain (32 kDa), which is an *N*-glycosidase, and a receptor-binding domain consisting of five identical B chains (about 7.7 kDa each). These B chains form a ring, and the A chain connects to the B chain by inserting its C-terminus into the center pore of the B chain ring. Stx1 has only one single amino acid difference from Stx, while Stx2 represents a distinct serotype, with about 56% sequence identity to Stx.

Ricin and Stxs also share similar entry pathways into cells [2,3,5,6]. Once they have entered cells through endocytosis, they are sorted into retrograde trafficking routes and enter the endoplasmic reticulum (ER) through the Golgi apparatus. Their A chains are then released from the ER into the cytosol, utilizing the host protein translocation machinery on the ER membranes. Consistent with this trafficking pathway, inhibitors that disrupt the Golgi apparatus, such as Brefeldin A, block ricin and Stxs toxicity. Small molecule inhibitors that disrupt the specific retrograde transport pathways utilized by ricin and Stxs have also been reported (Retro-1 and Retro-2) [7], although the host targets for Retro-1/2 remain to be established.

The major difference between ricin and Stxs is their receptor recognition. Ricin binds broadly to glycan moieties containing galactose and *N*-acetylgalactosamine [8]. In contrast, Stxs specifically recognize the glycan headgroup of Gb3 (also known as CD77), a glycosphingolipid [2,3,9]. Crystal structure studies suggest that each Stx B domain contains three potential Gb3 binding sites; thus, one Stx may simultaneously cluster up to 15 Gb3 molecules [10]. Expression of Gb3 is highly restricted in a subset of germinal center B cells, kidney tissues, vascular endothelial cells, and neurons, while the majority of other cell types do not express detectable levels of Gb3. Species such as cattle and deer do not express Gb3 and can serve as natural reservoirs for Shigatoxigenic bacteria [11].

Glycosylation is one of the most common modifications of proteins and membrane lipids [12,13]. It is initiated in the ER or on the ER membranes, while the majority of the remaining steps are carried out inside the Golgi apparatus. Transfer of sugar moieties to proteins or lipids are catalyzed by various glycosyltransferases (GTs) [14]. Genetic defects in protein and lipid glycosylation result in congenital disorders of glycosylation (CDG), a growing disease family comprising nearly a hundred disorders [15]. These defects occur not only on GTs but also on key regulatory proteins that control the specificity/activity of GTs. Toxins that utilize glycan moieties as their receptors could serve as powerful tools in mutagenesis screens to identify host proteins involved in glycosylation [12,13]. Indeed, ricin has undergone various genome-wide screens on mammalian cells, including small interfering ribonucleic acid (siRNA)-mediated knockdown (KD) approaches, Clustered Regularly Interspaced Short Palindromic Repeats (CRISPR)-Cas9–mediated KD and knockout (KO) approaches, and retroviral mutations in haploid cells [16–19]. These screens have yielded a list of GTs and nucleotide transporters (NTs) involved in *N*-linked protein glycosylation.

Here, we identified a human bladder cancer cell line that is highly sensitive to Stxs. Utilizing this cell line, we carried out CRISPR-Cas9–mediated KO screens for Stx1 and Stx2. In addition, we also carried out a genome-wide screen for ricin. These screens identified lysosomal-associated protein transmembrane 4 alpha (LAPTM4A) as a novel Golgi protein specifically required for Gb3 biosynthesis. The screens also revealed two Golgi proteins, Transmembrane protein 165 (TMEM165) and Transmembrane 9 superfamily member 2 (TM9SF2), as key factors for maintaining proper glycosylation levels in cells.

## Results

### Genome-wide CRISPR screens for Stx1 and Stx2

**Fig 1:**
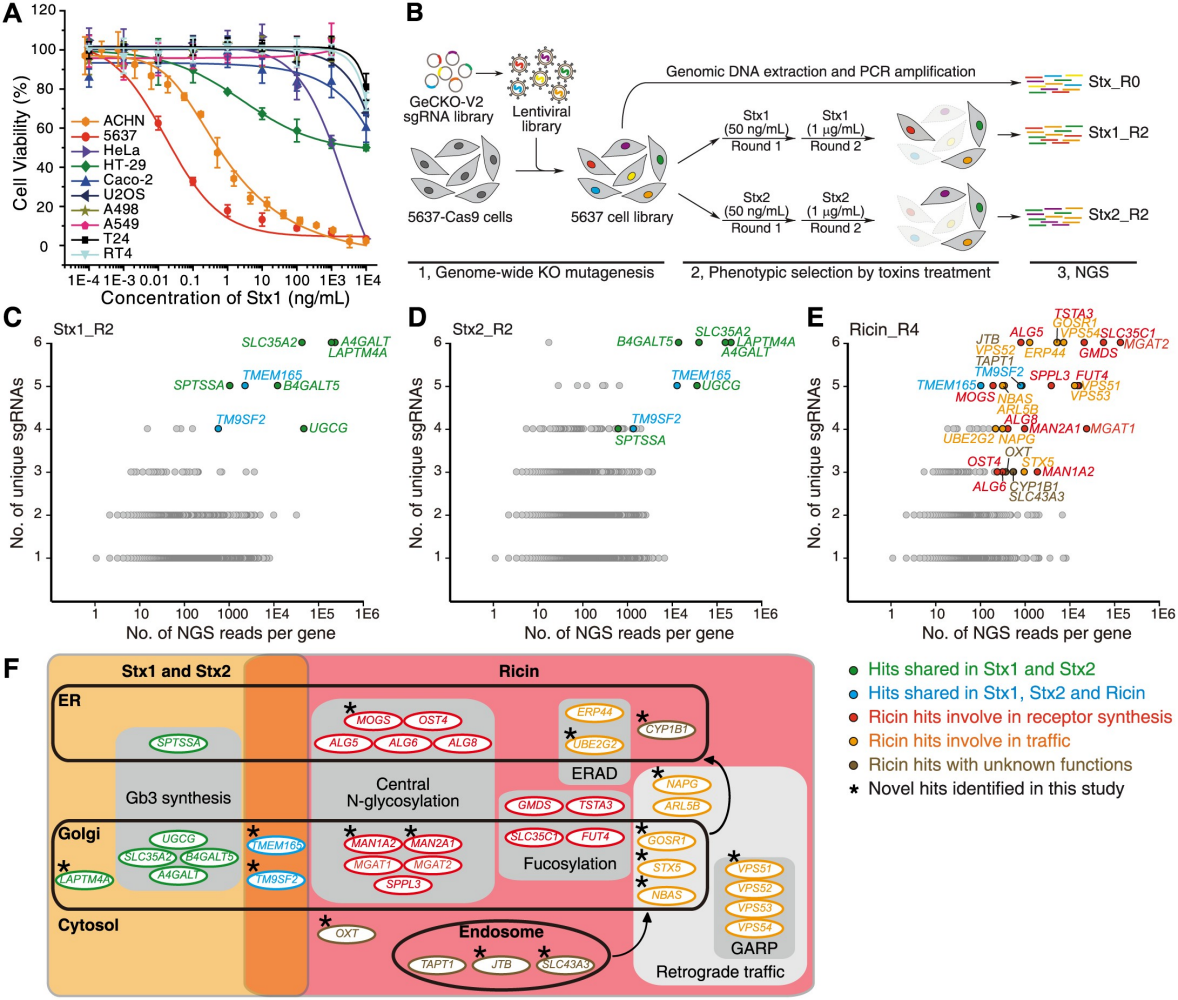
Genome-wide loss-of-function CRISPR-Cas9 screens to identify host factors for Stx1, Stx2, and ricin. (A) A range of human cell lines were exposed to a series of concentrations of Stx1 for 72 h and cell viability was measured using MTT assays. The toxin concentrations that resulted in the death of 50% of cells are defined as IC_50_ and listed in S1 Table. The bladder cancer cell line 5637 was the most sensitive one to Stx1, followed by the kidney cancer cell line ACHN. Error bars indicate mean ± SD, *N* = 3. (B) Schematic diagram of the screen process. Cells of cell line 5637 (hereafter, if the cell line is not specified, it is 5637 by default) that stably express Cas9 were transduced with lentiviral sgRNA libraries. These cells were then selected with Stx1 or Stx2 at 50 ng/mL for 48 h. The surviving cells were recovered in toxin-free medium and then subjected to the second round (R2) of selection with 1 μg/mL Stx1 or Stx2. The remaining cells were harvested and sgRNA sequences identified by NGS. Cells that were not exposed to toxins (Stx_R0) served as controls. (C-E) Genes identified in the screens with Stx1, Stx2, or ricin are ranked based on the number of unique sgRNAs (*y* axis) and total number of NGS reads (*x* axis). Top hits that overlap between Stx1 and Stx2 are marked in green. The two hits that appear across Stx1, Stx2, and ricin screens are marked in blue. The ricin-specific hits involved in receptor synthesis, membrane trafficking, and unknown functions are marked in red, orange, and brown, respectively. (F) The top hits marked in C–E were grouped by their subcellular localizations and the cellular pathways in which they might be involved. The asterisks mark novel hits identified in this study.

We first assessed the sensitivity of a panel of human cancer cell lines to Stx1 and Stx2 using a 72-h cell viability assay. The cells were exposed to a titration of toxins for 72 h, and the percentage of surviving cells was measured using MTT assays (Fig 1A and S1A Fig). The toxin dose that induced death of 50% cells is designated IC_50_. Most cell lines are insensitive to Stx1 and Stx2, with IC_50_ > 10,000 ng/mL (S1 Table). The bladder carcinoma cell line 5637 was the most sensitive one to Stx1 and Stx2, with IC_50_ at about 0.028 (Stx1) and 0.007 (Stx2) ng/mL. The human kidney adenocarcinoma cell line ACHN is also quite sensitive to Stx. This level of sensitivity is not shared by two other bladder cancer cell lines (T24 and RT4), suggesting that it is not a general feature of bladder cancer cells. Consistently, immunostaining analysis using a polyclonal anti-Stx1 antibody showed robust binding of Stx1 to 5637 and ACHN cells, whereas binding to HeLa cells was undetectable (S1D Fig).

Utilizing 5637 cells, we carried out genome-wide CRISPR-Cas9–mediated loss-of-function screens. Cells stably expressing Cas9 were established and transduced with a lentiviral sgRNA library (GeCKO V2), targeting 19,052 human genes with six single guide ribonucleic acids (sgRNAs) per gene [20,21]. These cells were then subjected to two rounds of selection with Stx1 or Stx2 (Fig 1B). The sgRNA sequences in surviving cells were identified via next-generation sequencing (NGS). Cells that were not treated with toxins served as controls. The identified genes were ranked based on the number of unique sgRNAs (*y* axis) and the total number of NGS reads (*x* axis) (Fig 1C and 1D). The full list of screen results is shown in S1 Data.

Most top-ranked hits overlapped between Stx1 and Stx2 screens. Five of the top eight genes are known factors in the Gb3 synthesis pathway (S1G Fig): *A4GALT*, *B4GALT5*, *SLC35A2*, *UGCG*, and *SPTSSA* [13]. Serine palmitoyltransferase small subunit A (SPTSSA) is a part of the serine palmitoyltransferase complex on ER membranes, which catalyzes the first and rate-limiting step in sphingolipid biosynthesis to generate ceramide (Cer). Cer is then transported to the Golgi. Ceramide glucosyltransferase (UGCG) catalyzes glucose onto Cer on the cytosol side of the Golgi, which generates glucosylceramide (GlcCer). UGCG may also flip GlcCer into the lumen side of the Golgi, where *β*-1,4-galactosyltransferase 5 (B4GALT5) then catalyzes the transfer of a galactose from UDP-galactose onto GlcCer to generate lactosylceramide (LacCer), which is the precursor for both globo-series and ganglio-series of glycosphingolipids. *α*-1,4-galactosyltransferase (A4GALT) finally produces Gb3 by catalyzing the transfer of galactose to LacCer [22,23]. A4GALT is ranked number 1 in the Stx1 screen and number 2 in the Stx2 screen based on NGS reads (S1 Data). SLC35A2 (UDP-galactose translocator) transports UDP-galactose from the cytosol into the lumen of the Golgi.

The other three of the top eight genes are *LAPTM4A*, *TMEM165*, and *TM9SF2*. LAPTM4A is a poorly characterized small four-pass membrane protein with unknown function, previously proposed to be localized mainly on lysosomes [24–27]. It is ranked as high as A4GALT in our screens (S1 Data). TMEM165 is a multi-pass transmembrane protein localized in the Golgi. Mutations in TMEM165 have been linked to CDG, and TMEM165 deficiency causes a defect in glycosylation, possibly because of dysregulation of Mn^2+^ hemostasis in the Golgi, as Mn^2+^ is an essential metal cofactor for many GTs [28–30]. TM9SF2 contains a large N-terminal domain and nine transmembrane domains; its function remains unknown [31]. Both *TMEM165* and *TM9SF2* have been previously identified as genes involved in heparan sulfate (HS) biosynthesis in two independent genetic screens using haploid cells, suggesting that TMEM165 and TM9SF2 deficiency affects multiple glycosylation pathways [32,33]. Interestingly, a recent genome-wide CRISPR-Cas9 screen, carried out by incubating EHEC with human colorectal carcinoma cell line HT-29, also identified LAPTM4A and TM9SF2 as key factors for EHEC toxicity to cells [34]. Furthermore, LAPTM4A KO and TM9SF2 KO cells showed no binding of Stx1, suggesting that the deficiency in LAPTM4A or TM9SF2 reduces Gb3 in cells [34].

To validate our screen results, we generated mixed (uncloned population) KO 5637 cells using the CRISPR-Cas9 approach for *A4GALT*, *SLC35A2*, *UGCG*, *B4GALT5*, *LAPTM4A*, *TMEM165*, and *TM9SF2*. Cell viability assays were carried out to determine their sensitivity to Stx1 and Stx2 (S1 Table). A4GALT, SLC35A2, UGCG, B4GALT5, and LAPTM4A KO cells all showed > 10^5^-fold increase in resistance to Stx1 and Stx2 compared to wild-type (WT) cells. TM9SF2 KO and TMEM165 KO cells showed about 10-fold and 780-fold smaller increases in resistance compared with LAPTM4A KO and A4GALT KO cells.

### Genome-wide CRISPR screen for ricin

In contrast to Stxs, most cells are sensitive to ricin (S1B Fig and S1 Table). We thus utilized HeLa cells that stably express Cas9 for a genome-wide screen for ricin. The sgRNA sequences in surviving cells were identified and ranked (Fig 1E and S2 Data). The majority of top-ranked genes fall into four pathways: *N*-linked protein glycosylation, fucosylation, membrane trafficking, and ER-associated protein degradation (ERAD)/quality-control pathways (Fig 1F). Top hits involved in glycosylation include enzymes located in the ER for catalyzing formation and degradation of high mannose oligosaccharide (*ALG5*, *ALG6*, *ALG8*, *MOGS*) and transferring oligosaccharides to an asparagine residue as an *N*-linked glycan (*OST4*), as well as enzymes located in the Golgi that convert oligo-mannose to galactose-containing complex *N*-linked glycans (*MAN1A2*, *MAN2A1*, *MGAT1*, and *MGAT2*). These results are consistent with the established view that galactose on *N*-linked glycans is the primary receptor for ricin.

Our screens also identified key players in the fucosylation pathway, including TSTA3 and GMDS, which catalyze the synthesis of GDP-fucose; SLC35C1, which transports GDP-fucose from the cytosol into the Golgi lumen; and FUT4, which catalyzes the transfer of fucose to *N*-acetyllactosamine to generate fucosylated carbohydrates, such as the non-sialylated carbohydrate antigen, Lewis X. The critical role of fucosylation in ricin toxicity has been previously reported and validated, possibly because fucosylation promotes the exposure of terminal galactoses by preventing their sialylation [19,35,36].

The screens revealed a series of proteins involved in intracellular vesicular transport, including members of the Golgi-associated retrograde protein (GARP) complex (VPS51, 52, 53, 54), which is a tethering complex involved in retrograde transport from the early endosome to the Golgi, as well as a few proteins involved in Golgi-ER trafficking (e.g., GOSR1, NBAS, STX5, NAPG, ARL5B). There are also two ER proteins among the top-ranked hits: ER resident protein 44 (ERP44), which is a member of the protein disulfide isomerase family, and ubiquitin conjugating enzyme E2 G2 (UBE2G2), which is an E2 ubiquitin-conjugating enzyme involved in ERAD.

Ricin has been previously subjected to shRNA-mediated KD screens and a CRISPR-Cas9–mediated loss-of-function screen utilizing a K562 cell line (a human bone marrow lymphoblast) and a retroviral mutagenesis screen using haploid cells [16–19]. Among our top 20 hits, 13 overlap with the top hits of at least one of the previous screens, providing a degree of validation across distinct cell lines [17,18]. Among the other seven newly identified hits, two (VPS51 and MOGS) are within well-established pathways required for ricin, as described above. The other five are GOSR1, JTB, NBAS, TMEM165, and TM9SF2. Golgi SNAP receptor complex member 1 (GOSR1) and neuroblastoma-amplified sequence (NBAS) are involved in Golgi-ER trafficking. Jumping translocation breakpoint protein (JTB) may play a role in regulating cell proliferation, but its role in ricin intoxication remains unknown. TMEM165 and TM9SF2 are the only two top-ranked factors shared between ricin and Stx screens.

To validate our ricin screen results, we generated mixed KO HeLa cells using the CRISPR-Cas9 approach for *MGAT2*, *SLC35C1*, *ERP44*, and *TAPT1*, which are among the top hits reported from previous screens but have not been experimentally validated. We also generated mixed KO cells for the three new factors GOSR1, JTB, and NBAS. These KO cells all showed modest increases (about 10–200-fold) in resistance to ricin compared with WT cells (S2E Fig and S1 Table).

### LAPTM4A KO cells lose binding of Stxs

We then focused on investigating the role of LAPTM4A, which is specific for Stxs, as well as TMEM165 and TM9SF2, the two factors shared between Stxs and ricin. We first examined whether the requirement of LAPTM4A is limited to 5637 cells. We generated LAPTM4A KO ACHN cells using the CRISPR-Cas9 approach, which became resistant to Stx1 and Stx2 (S2C and S2D Fig). To further address the concern on potential off-target effects, we generated a second line of LAPTM4A KO 5637 cells using a different sgRNA sequence (S2 Table). This new KO line was resistant to Stx1 and Stx2 as well (LAPTM4A-KO-II-Mix, S2A and S2B Fig). We also generated a 5637 KO cell line lacking LAPTM4B, which is a homolog of LAPTM4A. This cell line showed sensitivity to Stx1 and Stx2 similar to that of WT cells (S2A and S2B Fig), suggesting that the role of LAPTM4A is not shared by its homolog.

**Fig 2:**
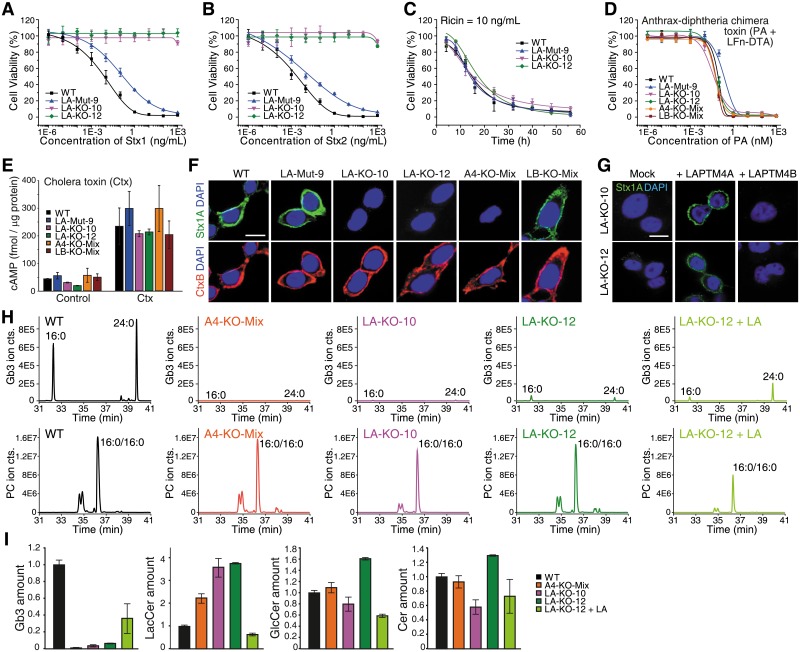
LAPTM4A KO cells lose binding of Stxs. (A-B) Two single clones of LAPTM4A KO 5637 cells (LA-KO-10 and LA-KO-12) are resistant to Stx1 and Stx2 in cell viability assays. One LAPTM4A mutant cell line (LA-Mut-9), which expresses a mutant form of LAPTM4A with three residues deleted (Met57, Pro58, Ala59), showed a lower sensitivity to Stx1 and Stx2 compared with WT cells. The IC_50_ numbers are listed in S1 Table. Error bars indicate mean ± SD, *N* = 3. (C) LA-KO-10 and LA-KO-12 cells showed levels of sensitivity to ricin (10 ng/mL) similar to those of WT cells. Error bars indicate mean ± SD, *N* = 3. (D) LA-KO-10 and LA-KO-12 cells showed sensitivity to A-Dtx similar to that of WT cells. Mixed stable KO cells lacking A4GALT (A4-KO-Mix) or LAPTM4B (a homolog of LAPTM4A, LB-KO-Mix) were examined in parallel; both showed sensitivity to A-Dtx similar to that of WT cells (Student’s *t* test based on comparing IC_50_ between WT and KO cells, *p* > 0.05). PA and LFn-DTA were added to the medium at a ratio of 4:1. The *x* axis shows the concentrations of PA. Cells were exposed to the toxin for 72 h and cell viability was measured using MTT assays. Error bars indicate mean ± SD, *N* = 3. (E) Cells were exposed to Ctx (50 μg/mL, 4 h), and cAMP levels in cell lysates were measured using a commercial kit. Cells that were not exposed to Ctx were analyzed in parallel as controls. LA-Mut-9, LA-KO-10, LA-KO-12, A4-KO-Mix, and LB-KO-Mix cells all showed sensitivity to Ctx similar to that of WT cells. (F) Cells were exposed to Stx1 (4.8 μg/mL) and CtxB (2 ng/mL) on ice for 60 min, washed, fixed, and subjected to immunostaining without permeabilization. Stx1 was detected using a polyclonal Stx1 antibody. CtxB was conjugated with Alexa555. Nuclei were labeled with DAPI. LA-KO-10, LA-KO-12, and A4-KO-Mix cells showed no binding of Stx1, while binding of CtxB to these cells was not changed. LA-Mut-9 and LB-KO-Mix were analyzed in parallel, showing no reduction in Stx1 binding. Scale bar, 5 μm. Representative images were from one of three independent experiments. (G) Expression of LAPTM4A, but not LAPTM4B, in LA-KO-10 and LA-KO-12 cells restored binding of Stx1. LAPTM4A and LAPTM4B were expressed via transient transfection. Scale bar, 5 μm. Representative images were from one of three independent experiments. (H) The levels of Gb3 in cells were quantified using mass spectrometry analysis. Ion chromatograms for Gb3 and phosphatidylcholine (PC) are shown using their respective protonated ion mass centered within 15 ppm for the most abundant fatty acyl chains (16:0 and 24:0) for Gb3 and for PC (16:0/16:0). Quantification of Gb3 was normalized with PC as an internal standard. Quantification data are listed in S4 Data. (I) Mass spectrometry analysis revealed that Gb3 is greatly reduced in LA-KO-10 and LA-KO-12 cells. A4-KO-Mix, which lacks Gb3 as well, was analyzed in parallel. The levels of LacCer, the precursor of Gb3, are elevated in all three Gb3-deficient cell lines, while the levels of GlcCer and Cer are similar to those in WT cells. Expression of LAPTM4A increased Gb3 and reduced LacCer levels in LA-KO-12 cells. Error bars indicate mean ± SD, *N* = 3.

To further investigate the role of LAPTM4A, we isolated single clones from the LAPTM4A KO population. The genotype of each clone was determined by sequencing, which indicates that 5637 cells contain three sets of chromosomes (S3 Data). Two lines (KO-10 and KO-12) contain frameshift mutations at the target region on all three chromosomes. One line (Mut-9) contains frameshift mutations on two chromosomes and a deletion of nine base pairs on the third chromosome. Consistent with these genotyping results, KO-10 and KO-12 cells are resistant to Stx1 and Stx2 in cell viability assays, while Mut-9 cells showed only reduced sensitivity to both toxins (Fig 2A and 2B and S3 Table).

To determine whether increased resistance is specific to Stxs, we examined the sensitivity of LAPTM4A KO cells to ricin and two other bacterial exotoxins: anthrax-diphtheria chimeric toxin (A-Dtx) and cholera toxin (Ctx). A-Dtx is composed of the N-terminal part of anthrax toxin lethal factor (LFn) fused to the enzymatic domain of diphtheria toxin (DTA) and the receptor-binding/translocation domains of Anthrax toxin (PA) [37]. PA mediates binding and entry of toxins into cells and translocates DTA into the cytosol from endosomes. DTA induces death of cells by inhibiting protein synthesis—the same process disrupted by ricin and Stxs, but the entry of this chimeric toxin does not require retrograde transport into the Golgi-ER. Ctx utilizes ganglioside GM1 as its major receptor and requires retrograde transport into the Golgi-ER for its release into the cytosol [38]. Ctx then catalyzes ADP-ribosylation of the Gs alpha subunit, which elevates cAMP levels. The sensitivity of cells to ricin and A-Dtx was determined by cell viability assays, while the sensitivity to Ctx was quantified by measuring cAMP levels in cell lysates. KO-10 and KO-12 showed sensitivity to ricin, A-Dtx, and Ctx similar to that of WT cells (Fig 2C–2E and S3 Table). We also examined Mut-9, A4GALT KO, and LAPTM4B KO cells, which all showed levels of sensitivity to these toxins similar to that of WT cells.

To determine which step of toxin action is affected in LAPTM4A KO cells, we first examined binding of Stx1 to cells via immunofluorescent staining. WT and Mut-9 cells showed robust binding of Stx1, while there was no detectable binding to KO-10 or KO-12 cells (Fig 2F). As expected, A4GALT KO cells showed no binding of Stx1, while LAPTM4B KO cells showed robust binding (Fig 2F). Furthermore, transfecting KO-10 and KO-12 cells with a plasmid that expresses LAPTM4A rescued binding of Stx1, while expression of LAPTM4B in these two KO cells did not restore Stx1 binding (Fig 2G). We also examined binding of Ctx utilizing a fluorescently labeled receptor-binding domain of Ctx (CtxB). Unlike Stx1, CtxB showed similar levels of binding to all these cells (Fig 2F), suggesting that lack of LAPTM4A specifically affects the Gb3 branch of glycolipids. In addition to immunofluorescent staining, binding of Stx1 was also analyzed by immunoblot of cell lysates, as well as by flow cytometry, which yielded the same results as immunofluorescent staining (S3 Fig).

### LAPTM4A KO cells lose Gb3 expression

Loss of Stx1 binding implies that LAPTM4A is critical for Gb3 expression. To examine this possibility directly, we sought to quantify the levels of Gb3 and its precursors LacCer, GlcCer, and Cer in cells using mass spectrometry analysis. Total lipids were extracted from cell lysates. Samples were then analyzed using an ultra-pressure liquid chromatograph (UPLC) coupled to a mass spectrometer. Quantification of glycolipids was normalized based on endogenous phosphatidylcholine (PC) as an internal standard (Fig 2H and S4A Fig). We found that Gb3 is greatly reduced in KO-10 and KO-12 cells, similar to the case in A4GALT KO cells (Fig 2I and S4 Data). Ectopic expression of LAPTM4A in KO-12 cells partially restored Gb3 levels. In contrast to Gb3, levels of LacCer are elevated in KO-10 and KO-12 cells, as well as in A4GALT KO cells. This increase in LacCer is abolished with expression of LAPTM4A in KO-12 cells. In addition, KO-12 cells also showed elevated GlcCer and Cer levels, both of which were restored with expression of LAPTM4A. These results demonstrate that LAPTM4A KO cells lack Gb3 expression. Because LacCer is elevated in LAPTM4A KO cells, it is likely that LAPTM4A is involved in Gb3 synthesis from LacCer, rather than in Gb3 degradation pathways.

### LAPTM4A is localized in the Golgi and interacts with A4GALT

**Fig 3:**
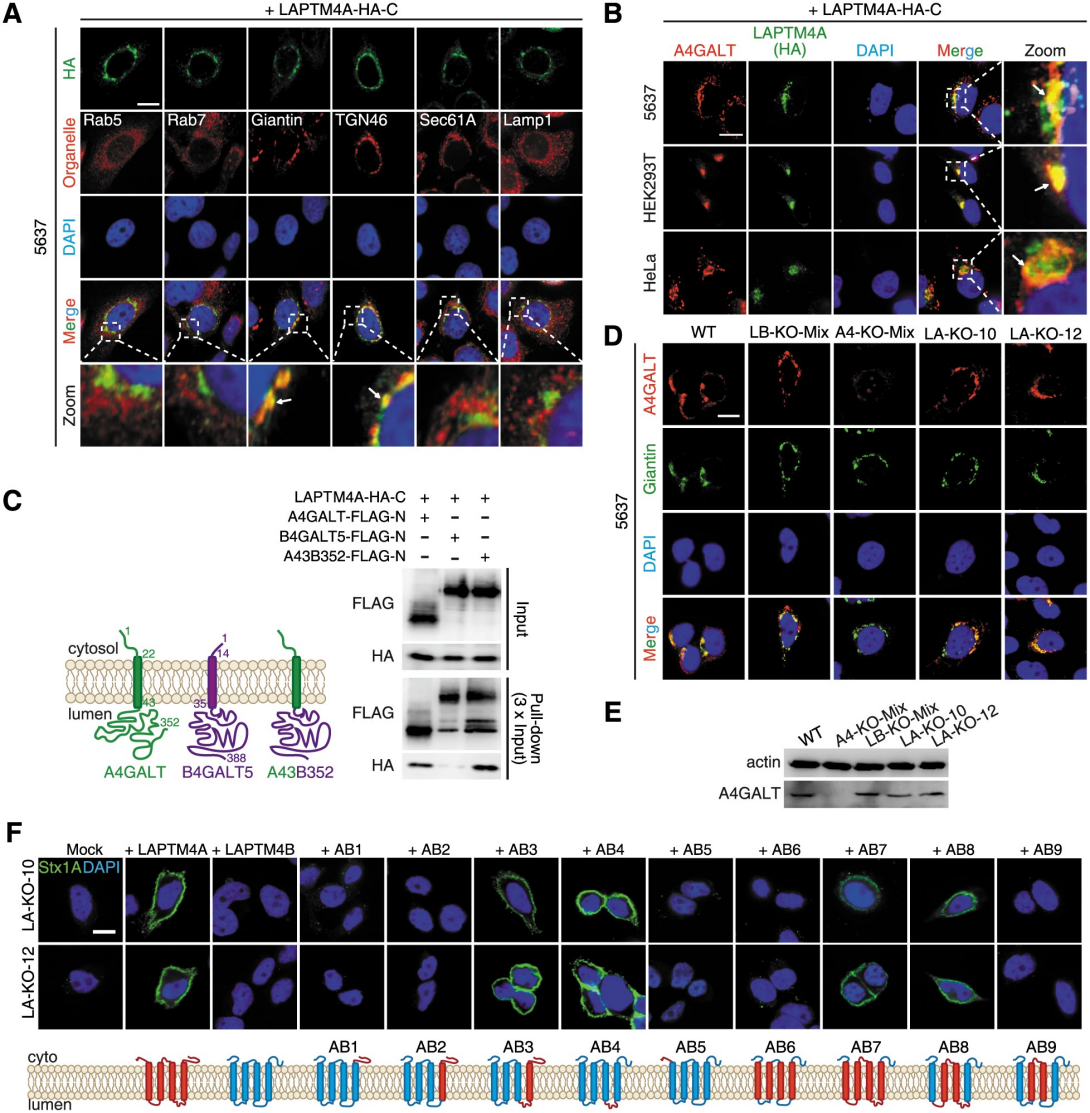
LAPTM4A forms a complex with A4GALT in the Golgi, and its second luminal region is critical for Gb3 biosynthesis. (A) LAPTM4A colocalizes with the Golgi markers in 5637 cells. LAPTM4A with a C-terminal triple HA tag was expressed in cells via transient transfection. Cells were fixed and subjected to immunofluorescent staining detecting the HA tag, as well as organelle markers: Rab5 (early endosomes), Rab7 (late endosomes), Giantin (Golgi), TGN46 (*Trans*-Golgi network), Sec61A (ER), and Lamp1 (lysosomes). (B) LAPTM4A colocalizes with endogenous A4GALT in 5637, HEK293T, and HeLa cells. LAPTM4A with a C-terminal triple HA tag was expressed in cells via transient transfection and was detected using an HA-tag antibody. Endogenous A4GALT was detected using a rabbit polyclonal antibody. (C) Co-IP experiments were carried out using anti-FLAG magnetic beads for HEK293T cells co-transfected with HA-tagged LAPTM4A and FLAG-tagged A4GALT, B4GALT5, or a chimeric protein (A43B352, composed of the cytosolic and transmembrane domain of A4GALT and luminal domain of B4GALT5). Samples were analyzed by immunoblot using anti-FLAG and anti-HA antibodies. LAPTM4A was co-immunoprecipitated with A4GALT, but not B4GALT5. A43B352 was also able to co-immunoprecipitate with LAPTM4A. The schematic drawings depict the topology of A4GALT, B4GALT5, and A43B352. (D) Endogenous A4GALT is expressed at similar levels and remains localized in the Golgi in two LAPTM4A KO cell lines (LA-KO-10 and LAKO-12) compared to WT and LAPTM4B KO cells (LB-KO-Mix). The specificity of A4GALT antibody is confirmed as it did not detect any signals in A4GALT KO cells (A4-KO-Mix). Giantin marks the Golgi. (E) Cell lysates of WT, A4-KO-Mix, LB-KO-Mix, LA-KO-10, and LA-KO-12 cells were subjected to immunoblot analysis detecting endogenous A4GALT. LA-KO-10 and LA-KO-12 express A4GALT similar to the levels seen in WT cells. One of two independent experiments is shown. (F) The second luminal domain of LAPTM4A is critical for Gb3 biosynthesis. A series of chimeric proteins (AB1–AB9) between LAPTM4A and LAPTM4B, as depicted in the schematic drawings, were expressed in LA-KO-10 and LA-KO-12 cells. Binding of Stx1 to these cells was examined by immunostaining. Replacing just the second luminal domain of LAPTM4B with the corresponding region in LAPTM4A resulted in a chimeric protein (AB4) that restored Stx1 binding. All scale bars, 5 μm. Arrows mark colocalization. Representative images are from one of three independent experiments.

To understand the function of LAPTM4A, we next examined its subcellular localization. As there is no suitable antibody to detect endogenous LAPTM4A, we utilized a LAPTM4A tagged with a triple HA tag on its C-terminus. This tagged version restored binding of Stx1 when expressed in KO-10 and KO-12 cells (Fig 2G). Expression of this tagged LAPTM4A in 5637, HEK293T, and HeLa cells all showed high degrees of colocalization with both Giantin and TGN46, two well-established markers for the Golgi, but not other organelle markers such as Rab5 (early endosome), Rab7 (late endosome), Sec61A (ER), or Lamp1 (lysosome) (Fig 3A and S5 and S12 Figs). We also examined localization of endogenous A4GALT using a polyclonal antibody whose specificity was confirmed using A4GALT KO cells (S6A Fig). As expected, A4GALT largely colocalizes with the Golgi marker Giantin. Consistently, HA-tagged LAPTM4A colocalizes with A4GALT in 5637, HEK293T, and HeLa cells (Fig 3B).

To further examine whether LAPTM4A interacts with A4GALT, we co-expressed HA-tagged LAPTM4A and FLAG-tagged A4GALT in HEK293T cells and then carried out co-immunoprecipitation (co-IP) assays using a FLAG tag antibody. The FLAG-tagged A4GALT is correctly localized to the Golgi and can rescue binding of Stx1 to A4GALT KO cells (S6B–S6D Fig). In addition, we also utilized a FLAG-tagged B4GALT5 as a control. Both A4GALT and B4GALT5 are type II transmembrane proteins in the Golgi, each with a single transmembrane domain and a short cytoplasmic domain located on its N-terminus. We found that LAPTM4A was specifically pulled down together with A4GALT, but not B4GALT5 (Fig 3C). Furthermore, a chimeric protein composed of the cytoplasmic and transmembrane domains of A4GALT and the luminal domain of B4GALT5 retained the ability to pull down LAPTM4A (Fig 3C).

We next analyzed whether the absence of LAPTM4A affects the Golgi localization and/or stability of A4GALT. We found that A4GALT is still localized to the Golgi in KO-10 and KO-12 cells and the expression levels of A4GALT in KO-10 and KO-12 cells are similar to those of WT and LAPTM4B KO cells (Fig 3D). Immunoblot analysis of the cell lysates further confirmed that the lack of LAPTM4A did not affect the level of A4GALT (Fig 3E). In addition, KO-10 and KO-12 cells also showed levels of A4GALT mRNA similar to that found in WT cells (S6E Fig).

LAPTM4A and LAPTM4B are small four-pass transmembrane proteins (S7A Fig) whose overall topology has not been established. We expressed two versions of LAPTM4A in cells, one with an HA tag on its N-terminus and the other with an HA tag on its C-terminus. We also utilized an ER membrane protein, ER membrane protein complex subunit 1 (EMC1), which has a single transmembrane domain, as a control. Cells were permeabilized with two different detergents: Digitonin, which permeabilizes only the plasma membrane but not the Golgi/ER membrane, or Saponin, which permeabilizes both the plasma membrane and the Golgi/ER membrane. Immunostaining analysis using an HA antibody labeled both the N-terminal-tagged and C-terminal-tagged LAPTM4A when cells were permeabilized with Digitonin (S7B Fig). In contrast, only C-terminal-tagged EMC1 was labeled, but not the N-terminal-tagged EMC1 under Digitonin treatment. These results suggest that both the N- and C-termini of LAPTM4A are exposed in the cytosol and that only two small regions (33 and 32 residues, respectively) between transmembrane domains are located within the Golgi lumen (S7C Fig).

Taking advantage of the observation that LAPTM4B cannot restore Stx1 binding to LAPTM4A KO cells, we generated a series of chimeric proteins between these two homologs in order to map the region responsible for their functional difference. These homologs were expressed in KO-10 and KO-12 cells and binding of Stx1 was analyzed by immunostaining, flow cytometry, and immunoblot (Fig 3F and S8 Fig). The results suggest that the second luminal domain (residues 129–159) is a critical region; engrafting this region to LAPTM4B generates a chimeric protein (designated AB4) that restored Stx1 binding. In contrast, engrafting other regions, such as the N-terminal cytosolic region, the C-terminal cytosolic region, the two middle transmembrane domains plus the middle cytoplasmic domain, or all four transmembrane domains, did not restore Stx1 binding. Co-IP analysis showed that both AB4 and LAPTM4B can interact with A4GALT (S6F Fig), suggesting that the interaction with A4GALT is mediated by regions conserved between LAPTM4A and LAPTM4B. We conclude that the second luminal domain accounts for the observed functional difference between LAPTM4A and LAPTM4B. It is likely that this luminal region of LAPTM4A contributes to Gb3 synthesis by influencing the catalytic activity/specificity of A4GALT, although the mechanism remains to be established.

### TMEM165 KO cells have lower levels of glycosphingolipids

**Fig 4:**
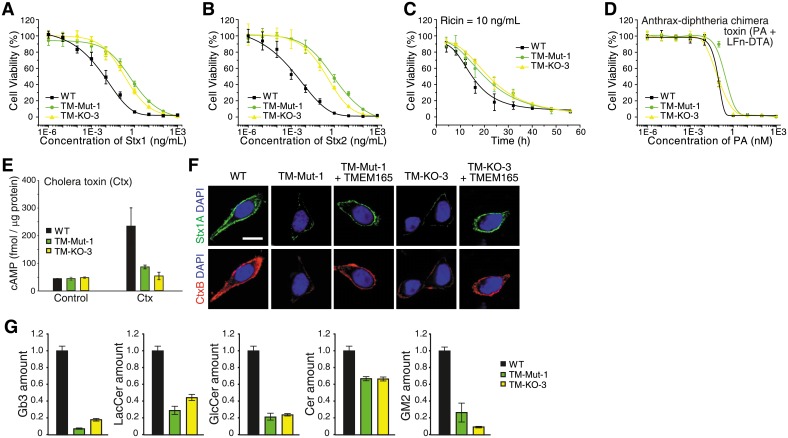
TMEM165 KO cells showed reduced levels of glycosphingolipids. (A-B) Both a TMEM165 KO cell line (TM-KO-3) and a mutant cell line (TM-Mut-1, insertion of Cys-Tyr residues between Leu279 and Cys280) showed increased resistance to Stx1 (A) and Stx2 (B). (C) TM-KO-3 and TM-Mut-1 cells showed reduced sensitivities to ricin (10 ng/mL) compared with WT cells. (D-E) TM-KO-3 and TM-Mut-1 cells showed similar sensitivities to A-Dtx (D) (Student’s *t* test, *p* > 0.05), but reduced sensitivity to Ctx (E) compared with WT cells. (F) TM-KO-3 and TM-Mut-1 cells showed reduced binding of Stx1 and CtxB. Ectopic expression of TMEM165 (with an N-terminal triple HA tag) restored binding of Stx1 and CtxB in both cell lines. Scale bar, 5 μm. Representative images are from one of three independent experiments. (G) Mass spectrometry analysis revealed that TM-KO-3 and TM-Mut-1 cells have lower levels of Gb3, LacCer, GlcCer, Cer, and gangliosides (GM2) compared with WT cells. Quantification data are provided in S4 Data. All error bars indicate mean ± SD, *N* = 3.

We next investigated the role of TMEM165 and generated single clones of TMEM165 KO 5637 cells via the CRISPR-Cas9 approach. We obtained one KO line (TM-KO-3) that contains frameshift mutations on all chromosomes, and one mutation line (TM-Mut-1) that contains a frameshift and an insertion of six base pairs (S3 Data). The latter still expressed TMEM165, but with two extra residues inserted at the target region (insertion of Cys-Tyr residues between Leu279 and Cys280). Cell viability assays showed that both TM-KO-3 and TM-Mut-1 cells are about 20–80-fold less sensitive to Stx1 and Stx2 compared with WT cells (Fig 4A and 4B and S3 Table).

To analyze the sensitivity of TMEM165-deficient cells to ricin, we initially carried out the standard 72-h cell viability assay, but the IC_50_ values were not significantly changed under our assay conditions (S3 Table). We then analyzed the sensitivity of these cells by fixing the ricin concentration and monitoring cell viability over time (Fig 4C). Both TM-Mut-1 and TM-KO-3 cells showed higher levels of surviving cells compared with WT cells when exposed to ricin for 20–30 h, and this difference disappeared by 40–50 h (Fig 4C), suggesting that TMEM165 deficiency caused a rather minor reduction in sensitivity to ricin. We also found that TMEM165-deficient cells showed similar levels of sensitivity toward A-Dtx but reduced sensitivity to Ctx, compared with WT cells (Fig 4D and 4E).

We next examined binding of Stx1 and CtxB to TMEM165-deficient cells by immunofluorescent staining, immunoblot, and flow cytometry (Fig 4F and S9A and S9B Fig). Both TM-KO-3 and TM-Mut-1 showed reduced binding of Stx1 and CtxB compared with WT cells, suggesting that a lack of TMEM165 affects the toxin binding step. Ectopic expression of TMEM165 restored binding of Stx1 and CtxB to TM-KO-3 and TM-Mut-1 cells (Fig 4F). Mass spectrometry analysis confirmed that both TM-KO-3 and TM-Mut-1 cells have lower levels of Gb3 compared with WT cells (Fig 4G and S4 Data). In contrast to LAPTM4A KO cells, these two lines showed lower levels of LacCer, GlcCer, and Cer. We further analyzed the levels of gangliosides in these TMEM165-deficient cells by mass spectrometry (S4B Fig) and found that TM-KO-3 and TM-Mut-1 cells showed low levels of gangliosides such as GM2 (Fig 4G), suggesting that the absence of TMEM165 affects the biosynthesis of glycosphingolipids globally.

### Defects in TMEM165 KO cells can be rescued by manganese

**Fig 5:**
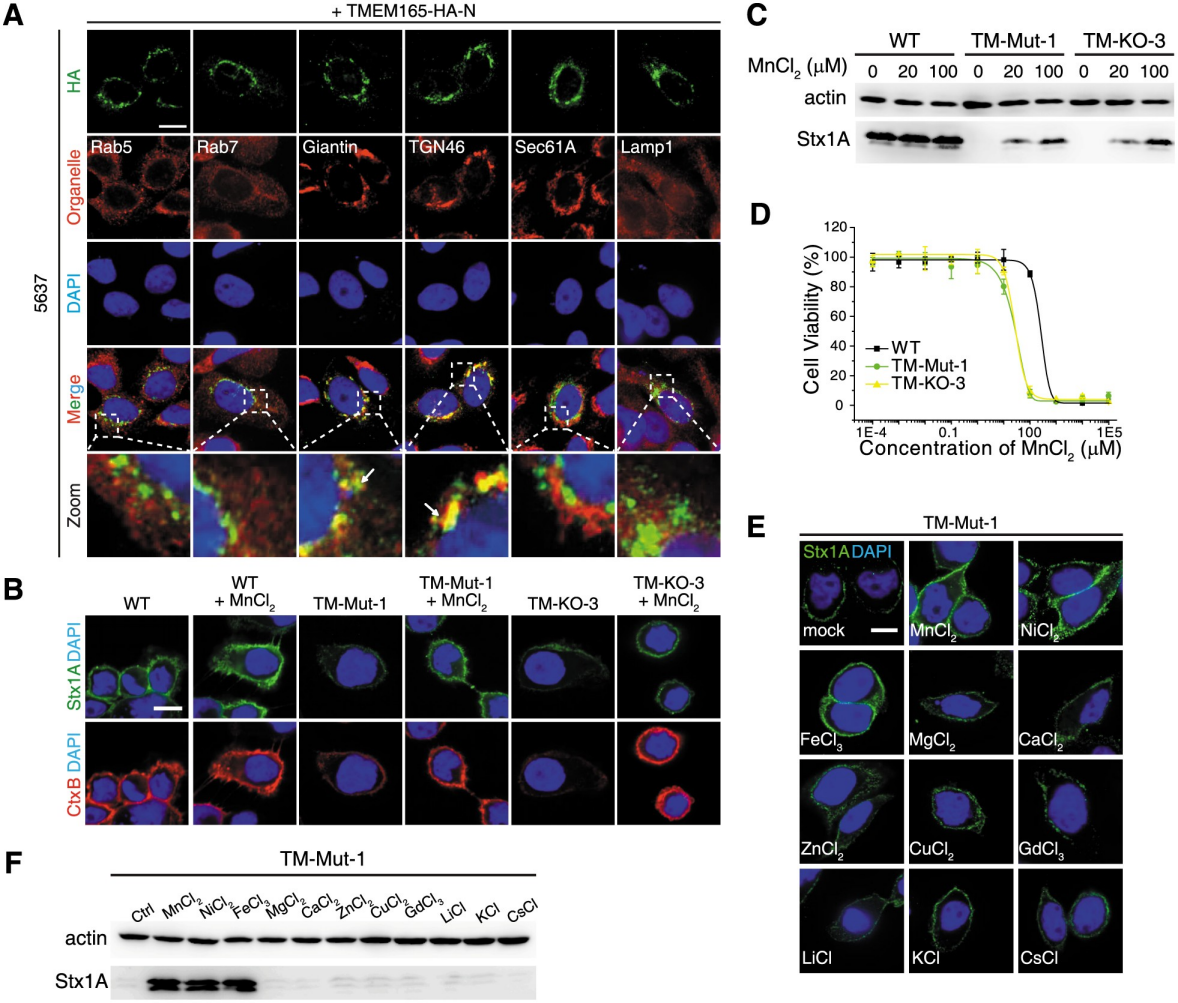
Adding manganese into medium restores binding of Stx1 to TMEM165-deficient cells. (A) HA-tagged TMEM165 colocalizes with Giantin and TGN46 in 5637 cells. (B) Pretreatment of TM-Mut-1 and TM-KO-3 cells with MnCl_2_ (100 μM, overnight) restored binding of Stx1 and CtxB to cell surfaces. (C) Binding of Stx1 to TM-Mut-1 and TM-KO-3 cells pretreated with MnCl_2_ (20 and 100 μM, overnight) was examined by immunoblot analysis, using a polyclonal Stx1 antibody. Actin served as a loading control. One of two independent experiments is shown. MnCl_2_ did not affect binding of Stx to WT cells but restored binding of Stx1 to TM-Mut-1 and TM-KO-3 cells. (D) WT, TM-Mut-1, and TM-KO-3 cells were exposed to a series of concentrations of MnCl_2_ for 3 d. Cell viability was determined using MTT assays. TM-Mut-1 and TM-KO-3 cells show greater sensitivity to the toxicity of MnCl_2_ compared with WT cells (Student’s *t* test, *p* = 0.012). Error bars indicate mean ± SD, *N* = 3. (E-F) Immunofluorescence analysis (E) and immunoblot analysis (F) were carried out to examine metal specificity. TM-Mut-1 cells pretreated with or without a panel of metal chlorides (100 μM, overnight) were subjected to toxin surface-binding assay. MnCl_2_, FeCl_3_, and NiCl_2_ restored binding of Stx1 to cell surfaces. Scale bar, 5 μm. Arrow, colocalization. Representative images were from one of three independent experiments.

Mutations in TMEM165 have been linked to a subtype of CDG. It has been reported that TMEM165 is localized to the Golgi and that the hypo-glycosylation defect in TMEM165-deficient cells can be rescued by supplementing the culture medium with Mn^2+^ [28–30]. Consistent with these reports, we found that TMEM165 colocalizes with the Golgi markers Giantin and TGN46 in 5637, HEK293T, and HeLa cells (Fig 5A and S9C, S9D and S12 Figs). Adding MnCl_2_ into medium partially restored binding of Stx1 and CtxB to TM-KO-3 and TM-Mut-1 cells (Fig 5B and 5C). Higher concentrations of Mn^2+^ are toxic to cells, and TM-KO-3 and TM-Mut-1 cells both showed a lower tolerance for high concentrations of Mn^2+^ compared with WT cells, further supporting the role of TMEM165 in regulating Mn^2+^ homeostasis (Fig 5D). We further screened other major metal ions and found that both NiCl_2_ and FeCl_3_ can also restore binding of Stx1 to TMEM165-deficient cells (Fig 5E and 5F).

### TM9SF2 KO cells showed low levels of glycosphingolipids

**Fig 6:**
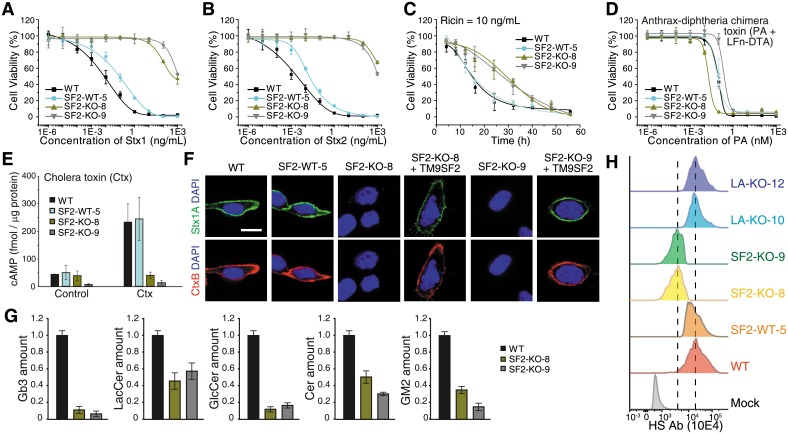
TM9SF2 KO cells showed reduced levels of glycosphingolipids. (A-B) TM9SF2 KO cells (SF2-KO-8 and SF2-KO-9) showed increased resistance to Stx1 (A) and Stx2 (B). SF2-WT-5 cells showed slightly reduced sensitivity to Stx1 and Stx2 compared with WT cells. Error bars indicate mean ± SD, *N* = 3. (C) SF2-KO-8 and SF2-KO-9 both showed lower sensitivities to ricin (10 ng/mL) compared with WT cells. (D-E) SF2-KO-8 and SF2-KO-9 cells showed similar sensitivities to A-Dtx (D) (Student’s *t* test, *p* > 0.05) but lower sensitivity to Ctx (E) compared with WT cells. (F) SF2-KO-8 and SF2-KO-9 cells showed reduced binding of Stx1 and CtxB. Ectopic expression of TM9SF2 (with a C-terminal triple HA tag) restored binding of Stx1 and CtxB in both cell lines. Scale bar, 5 μm. Representative images are from one of three independent experiments. (G) Mass spectrometry analysis revealed that SF2-KO-8 and SF2-KO-9 cells have lower levels of Gb3, LacCer, GlcCer, Cer, and GM2 compared with WT cells. Quantification data are listed in S4 Data. (H) SF2-KO-8 and SF2-KO-9 cells showed reduced levels of HS on cell surfaces, as measured by flow cytometry using an anti-HS antibody (10E4). LA-KO-10 and LA-KO-12 cells were also tested as controls. All error bars indicate mean ± SD, *N* = 3.

To examine the role of TM9SF2, two single KO 5637 cell lines were generated via the CRISPR-Cas9 approach. Both (SF2-KO-8 and SF2-KO-9) contain frameshift mutations in all three chromosomes (S3 Data). A single clone (SF2-WT-5) that still contains a WT allele was also selected as an additional control. Cell viability assays showed that both KO lines became highly resistant to Stx1 and Stx2, while SF2-WT-5 cells showed only a slight reduction in sensitivity compared with WT cells (Fig 6A and 6B and S3 Table). Similar to TMEM165 KO cells, the IC_50_ of TM9SF2 KO cells toward ricin was not significantly changed under our standard 72-h cell viability assay (S3 Table), but these cells showed greater viability after exposure to a fixed concentration of ricin for a shorter period of time (Fig 6C). TM9SF2 KO cells also showed no change in sensitivity to A-Dtx, but reduced sensitivity to Ctx (Fig 4D and 4E).

We found that binding of Stx1 and CtxB was largely abolished in two TM9SF2 KO cells, but not in the SF2-WT-5 control cells (Fig 6F and S10 Fig). Binding was restored in two KO cell lines when TM9SF2 was expressed via transient transfection (Fig 6F). Mass spectrometry analysis revealed that both TM9SF2 KO cells had low levels of Gb3, LacCer, GlcCer, Cer, and GM2 (Fig 6G), indicating that TM9SF2 deficiency caused global disruption in glycosphingolipid synthesis. It has been previously reported that TM9SF2 deficiency in haploid cells reduces HS biosynthesis [32,33]. Consistently, we found that surface HS levels were reduced in the two TM9SF2 KO cells when analyzed by flow cytometry using an antibody that recognizes HS (Fig 6H). These results confirmed that TM9SF2 deficiency affects multiple glycosylation pathways.

### TM9SF2 is localized in the Golgi

**Fig 7:**
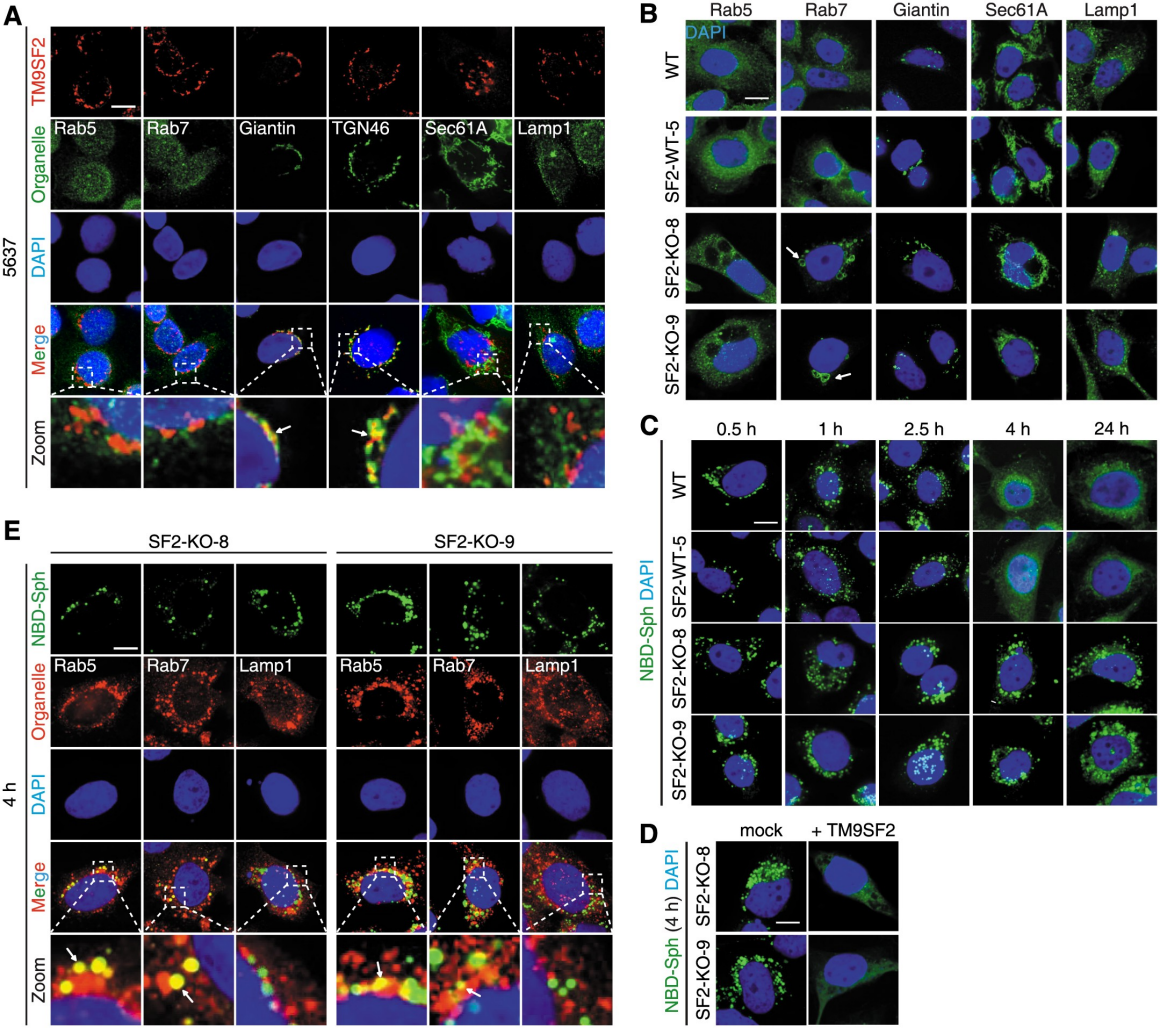
TM9SF2 is localized in the Golgi, and its deficiency disrupts endosomal trafficking. (A) Immunostaining analysis showed that endogenous TM9SF2 is colocalized with the Golgi markers Giantin and TGN46 in 5637 cells. TM9SF2 was detected using a chicken polyclonal antibody. (B) WT, SF2-WT-5, SF2-KO-8, and SF2-KO-9 were subjected to immunofluorescent staining with organelle markers Rab5, Rab7, Giantin, Sec61A, and Lamp1. Rab7 staining marked abnormally enlarged vesicular structures (marked by arrows) in SF2-KO-8 and SF2-KO-9 cells. (C-D) WT, SF2-WT-5, SF2-KO-8, and SF2-KO-9 cells were loaded with NBD-labeled sphingosine (NBD-Sph) on ice and then incubated at 37 °C for the indicated time. Internalized NBD-Sph was initially localized in endosomes and then dispersed throughout the cells by 4 h. In contrast, NBD-Sph remained in vesicular structures even at 24 h in SF2-KO-8 and SF2-KO-9 cells. This defect was rescued by ectopic expression of TM9SF2 (D). (E) SF2-KO-8 and SF2-KO-9 cells were loaded with NBD-Sph on ice and then cultured at 37 °C for 4 h. The internalized NBD-Sph colocalizes with Rab5 and Rab7, but not Lamp1. Arrows indicate colocalization. All scale bars, 5 μm. Representative images are from one of three independent experiments.

Previous studies have showed that Myc-tagged TM9SF2 is localized mainly on endosomes [31]. More recently, Tanaka and colleagues showed that endogenous TM9SF2, detected with a polyclonal TM9SF2 antibody, is localized in the Golgi in a haploid cell line [33]. Golgi localization was also recently confirmed for TM9SF2 in HeLa cells [34]. We first validated the specificity of this polyclonal antibody, which showed no signal on TM9SF2 KO cells (S11A Fig). We found that endogenous TM9SF2 in 5637, HEK293T, and HeLa cells are largely colocalized with the Golgi markers (Fig 7A and S11B, S11C and S12 Figs), confirming that TM9SF2 is a Golgi protein across multiple cell lines.

### TM9SF2 KO showed abnormal endosomal trafficking

Interestingly, both SF2-KO-8 and SF2-KO-9 cells appear to form many large vacuole-like structures within the cytosol, which are labeled with Rab7 (Fig 7B). To further examine endosomal trafficking in SF2 KO cells, we loaded exogenous sphingosine (Sph) labeled with the fluorescent dye nitrobenzoxadiazole (NBD). Sph was taken up by cells, trafficked through endosomes, and dispersed throughout the cells within 4 h in WT cells (Fig 7C). In contrast, trafficking of Sph was interrupted and remained within vesicular structures even at 24 h in both SF2-KO-8 and SF2-KO-9 cells (Fig 7C). This trafficking defect was rescued when TM9SF2 was expressed via transient transfection in SF2-KO-8 and SF2-KO-9 cells (Fig 7D). These Sph-containing vesicles are labeled with Rab5 and Rab7, but not Lamp1, suggesting that Sph was largely retained within endosomes in TM9SF2 KO cells (Fig 7E). We further examined trafficking of NBD-labeled Cer and phosphatidylserine (PS). Both lipids were taken up by cells and eventually dispersed within cells in WT cells. In contrast, both accumulated in vesicular structures in SF2-KO-8 and SF2-KO-9 cells (S11D and S11E Fig). Furthermore, we generated mixed TM9SF2 KO HeLa cells, which showed similar defects in trafficking of NBD-labeled Sph, Cer, and PS (S11F Fig), demonstrating that these trafficking defects exist across distinct cell types. These findings suggest that the absence of TM9SF2 not only disrupts glycosylation in Golgi but also severely disrupts endosomal trafficking in general, which may also contribute to resistance to Stxs and ricin.

## Discussion

Here, we found that the human bladder cancer cell line 5637 is highly sensitive to Stxs. Cell line 5637 is easy to culture and well suited for cell biology studies; thus, it can serve as a useful human cell model for studying Stxs. The top eight hits from Stx1 and Stx2 screens are all involved in Gb3 synthesis and cells lacking Gb3 are highly resistant to Stxs (10^5^-fold), further demonstrating that binding to Gb3 is the key rate-limiting step for Stx intoxication. In contrast, top hits from the ricin screen are distributed across multiple pathways, including protein glycosylation, fucosylation, retrograde trafficking, and ERAD pathway. Mutations in these ricin host factors elicited only low levels of resistance (<200-fold). These results are consistent with the view that various galactose-containing moieties and multiple trafficking pathways can mediate entry of ricin redundantly.

Other notable top-ranked hits shared between Stx1 and Stx2 screens include *ARCN1*, *UGP2*, and *SPPL3*. Coatomer subunit delta (ARCN1) is a component of the coat protein complex I (COPI) complex, a protein complex that mediates budding of vesicles from the Golgi membranes. COPI plays a critical role in vesicular transport between the Golgi and the ER [39], but its role for Stxs remains to be validated [17,40]. UDP-Glucose Pyrophosphorylase 2 (UGP2) is a cytosolic enzyme that produces UDP-glucose, a substrate required for glycosylation. Signal peptide peptidase-like 3 (SPPL3) is a membrane aspartic protease localized in the Golgi. It has been shown that SPPL3 cleaves many GTs and glycosidases within the Golgi, thus releasing their enzymatic domains into the Golgi lumen [41,42]. SPPL3 was identified in our ricin screen as well, although its exact role in relation to Stxs and ricin remains to be validated.

Our screening protocol relies on the cell viability assay after 72 h incubation. Such a long incubation could mask minor reductions in toxin trafficking within cells. It is possible that a few trafficking-related factors, such as GARP and STX5, were identified because they potentially may also affect glycosylation processes. Our Stx1 screen did not identify GPP130, a protein previously reported to be important for retrograde transport of Stx1 (but not Stx2) [43]. However, the role of GPP130 for Stxs remains to be established [44]. A recent shRNA-based KD screen for Stxs, utilizing HeLa cells that overexpress A4GALT, reported that defects in the Golgi protein UNC50 reduces the sensitivity of cells to Stx2 by about 200-fold [45]. UNC50 was not identified in our screen for Stx2.

Our major finding is identification of LAPTM4A, a poorly characterized small four-pass transmembrane protein, as a novel factor critical for Gb3 biosynthesis. Endogenous LAPTM4A protein has been detected in both the Golgi and lysosome fractions of rat liver membrane lysates [24,25]. Previous studies suggested that LAPTM4A is localized on late endosomes and lysosomes based on using LAPTM4A tagged with fluorescent protein mCherry, HA, or Myc on its N-terminus [25–27]. On the other hand, LAPTM4A tagged with the green fluorescent protein (GFP) on its C-terminus was shown to be localized in the Golgi in HeLa cells [34]. Here, we found that LAPTM4A, with a C-terminal HA tag, is primarily localized in the Golgi in three different cell lines.

The exact function of LAPTM4A remains to be established. It has been shown that LAPTM4A expression in yeast confers a multidrug-resistance phenotype and altered subcellular distribution of steroids, suggesting that LAPTM4A is involved in the transport of nucleosides or some small molecules [24,25,46]. LAPTM4B is up-regulated in many human cancer cells and has been suggested to be involved in recruitment of the leucine transporter to lysosomes, regulation of epidermal growth factor receptor lysosomal sorting and degradation, and facilitating Cer removal from late endosomes [47–49]. Despite the homology between LAPTM4A and LAPTM4B, only LAPTM4A is required for Gb3 synthesis. By switching different domains between LAPTM4A and LAPTM4B, we showed that the specificity is encoded within the second luminal domain of LAPTM4A.

Many GTs require other proteins for their stability/trafficking/activity. For instance, the core 1 β3galactosyltransferase (C1GALT1) requires the ER-localized membrane protein Cosmc (C1GALT1C1) as a molecular chaperone, which is essential for its proper folding and trafficking [50–52]. However, LAPTM4A does not appear to be required for stability/trafficking of A4GALT, as the Golgi-localization and expression levels of A4GALT are not affected in LAPTM4A KO cells.

The soluble enzymatic domain of GTs involved in glycolipid biosynthesis often showed no activity in vitro, suggesting that their activity requires a proper membrane environment and/or “activator” proteins to present the lipid acceptor to GTs. This requirement of an “activator” might be because the headgroups of lipid acceptors are difficult for the soluble enzymatic domain of GTs to reach. This is analogous to the well-established finding that degradation of sphingolipid requires cofactor proteins in addition to glycosidases. These cofactors are known as sphingolipid activator proteins, which are small enzymatically inactive proteins that present glycolipid substrates to glycosidases [53]. This activator function is similar to the previously proposed “add-on” domain for GTs [54]. For instance, it has been shown that *α*-lactalbumin can serve as an “add-on” domain for *β*4-galactosyltransferase, and formation of the complex changes the specificity of the acceptor [55]. We propose that LAPTM4A may act as an activator specifically for A4GALT, and that its second luminal domain facilitates recognition of the glycolipid substrates by A4GALT. A similar case is that of *Drosophila β*-1,4-*N*-acetylgalactosaminyltransferase B, which is responsible for synthesis of glycolipids and requires a six-pass membrane protein GABPI for its activity [56,57]. Furthermore, the two short luminal domains of GABPI (12 and 35 amino acids long) are required for the activity of this GT. GABPI has no mammalian homologs. LAPTM4A thus represents the first potential activator for glycolipid GTs identified in mammals, suggesting that the requirement of an activator could be a conserved feature for glycolipid GTs. GABPI is a member of the DHHC protein family, which are palmitoyltransferases, but it does not possess palmitoyltransferase activity, suggesting that it is utilized as a GT “activator” independent of its evolutionary origin [56]. It is possible that LAPTM4A has other functions independent of serving as an “activator” for A4GALT, as LAPTM4A is widely expressed across different tissues.

Our screens also identified TMEM165 and TM9SF2 as two host factors required for both Stx and ricin intoxication. TMEM165 has been proposed to maintain Mn^2+^ homeostasis within the Golgi, although its exact function remains to be fully established. Mn^2+^ is an essential cofactor for many Golgi GTs involved in both glycoprotein and glycolipid synthesis. It has been shown that TMEM165 deficiency leads to severe reduction in galactose and GalNAc in both glycoproteins and glycolipids [29]. This reduction can be rescued fully by Mn^2+^ and partially by galactose supplementation [29,58]. Consistently, we found that Mn^2+^ supplementation restored binding of Stx to TMEM165 KO cells. Furthermore, TMEM165 KO cells become more sensitive to toxicity from high concentrations of Mn^2+^. Together, these findings further support that TMEM165 plays a critical role in Mn^2+^ homeostasis in the Golgi.

The function of TM9SF2 remains a mystery. TM9SF2 is expressed in all tissues and is evolutionarily conserved. It belongs to a family of highly conserved membrane proteins, with three members in yeast (Tmn1 to Tmn3), *Drosophila* (TM9SF2 to TM9SF4), and *Dictyostelium discoideum* (Phg 1A to 1C) and four members in mammals (TM9SF1 to TM9SF4). The sequence features of this family suggest that they could function as channels or small-molecule transporters. Defects in TM9SF2 family members have been associated with two types of defects: (1) disruption in phagocytosis and endosomal trafficking: it has been shown that the absence of Phg 1A strongly impaired both cell adhesion and phagocytosis of bacteria in *Dictyostelium*, and both TM9SF4 and TM9SF2 are required for phagocytosis in *Drosophila* as well [59,60]. Furthermore, deletion of Tmn family members in yeast has also resulted in disruption of endosomal sorting [61]. Consistent with these results, we found that TM9SF2 KO cells showed defective endosomal trafficking. (2) Altering metal ion homeostasis: it has been reported that deletion of all three yeast homologs resulted in a 75% reduction in cellular copper (Cu) and a 50% increase in cellular Mn^2+^ [62]. This disruption in metal ion homeostasis may affect the glycosylation process in the Golgi, reducing the amount of both glycoproteins and glycolipids. Consistently, we found that TM9SF2 KO cells showed low levels of glycosphingolipids. The Golgi location of TM9SF2 suggests that the observed defects in endosomal trafficking might be an indirect effect, although it remains possible that TM9SF2 is also distributed at low levels on endosomes and is required for endosomal trafficking directly.

Globo-series glycosphingolipids are unique in that their expression is highly restricted. Our studies revealed that biosynthesis of Gb3 not only requires its GT but also a membrane protein, LAPTM4A, which may provide an additional layer of regulation to ensure the restricted expression of Gb3. Our studies also revealed two Golgi-localized factors required for maintaining a proper environment for GT activities within the Golgi. These findings further indicate that the glycosylation process in the Golgi is tightly controlled and regulated. Further research into the function of these factors will contribute to our understanding of the glycosylation process and CDG. Finally, our findings have also provided new potential therapeutic targets for treating Stxs and ricin intoxication.

## Materials and methods

### Antibodies, cell lines, and other reagents

The following reagents were purchased from commercial vendors: ricin (Vector Laboratories, L-1090, Burlingame, CA), Ctx (List Biological Laboratories, #101C, Campbell, CA), CtxB Alexa Flour 555 conjugate (Invitrogen, C34776, Waltham, MA), MTT (Sigma, M5655, St. Louis, MO), NBD-Sph (Avanti, 810205, Alabaster, AL), NBD-C6-Cer (Abcam, ab144090, Cambridge, MA), and NBD-PS (Avanti, 810192, Alabaster, AL). Antibodies for the following antigens were obtained from the indicated vendors: the A domain of Stx1 (Stx1A, List Biological Laboratories, #761L, Campbell, CA), Actin (Aves Labs, ACT-1010, Tigard, OR), HA-tag (BioLegend, 901502, San Diego, CA), FLAG-tag (Sigma, F3165, St. Louis, MO), Rab5 (Abcam, ab13253), Rab7 (Abcam, ab137029), Giantin (Abcam, ab80864 and ab37266), TGN46 (Abcam, ab50595), Sec61A (Abcam, ab183046), Lamp1 (Abcam, ab24170), and A4GALT (Abcam, ab98998). A chicken polyclonal anti-TM9SF2 antibody was generously provided by Dr. Yusuke Maeda (Osaka, Japan) [33]. The following cell lines were all originally obtained from ATCC with their catalog number noted: ACHN (CRL-1611), HeLa (CCL-2), HT-29 (HTB-38), Caco-2 (HTB-37), U2OS (HTB-96), A498 (HTB-44), A549 (CRM-CCL-185), 5637 (HTB-9), T24 (HTB-4), RT4 (HTB-2), and HEK293T (CRL-3216).

### cDNA constructs

Shiga toxin clones (pLPSH3 for Stx1 and pJES120 for Stx2) were generously provided by Dr. Alison O’Brien (Bethesda, MD). Protective antigen (PA) of Anthrax toxin was cloned into pET21a vector (Novagen) with a His6-tag at C-terminal. pET15b-LFn-DTA was obtained from Addgene (#11075). Recombinant PA and LFn-DTA were expressed in *E*. *coli* (BL21 strain) and purified as His-tagged proteins. The cDNAs of LAPTM4A (2958870), A4GALT (3913851), LAPTM4B (5264567), TMEM165 (8143981), TM9SF2 (40146324), EMC1 (4831005), and B4GALT5 (30915234) were purchased from GE Dharmacon. SgRNA-resistant full-length LAPTM4A, LAPTM4B, TM9SF2, and EMC1 with triple-HA tag at their C-termini (with EFGSGSGS as linker); full-length LAPTM4A, TMEM165, and EMC1 with triple-HA tag at their N-termini (with GSGSGSEF as linker); full-length A4GALT and B4GALT5 with triple-FLAG tag at their N-termini (with GSGSGSEF as linker) were cloned into ether pcDNA3.1 vector (Invitrogen, V800-20) or pLenti-Hygro vector (Addgene, #17484). The LAPTM4A (LA)-LAPTM4B (LB) chimeric proteins were constructed as the following: AB1 (LB_1–173_–LA_181–233_), AB2 (LB_1–153_–LA_161–233_), AB3 (LB_1–120_–LA_129–233_), AB4 (LB_1–120_–LA_129–160_–LB_154–226_), AB5 (LA_1–29_–LB_26–226_), AB6 (LB_1–25_–LA_30–49_–LB_47–71_–LA_83–102_–LB_93–99_–LA_109–128_–LB_121–152_–LA_161–180_–LB_174–226_), AB7 (LB_1–25_–LA_30–180_–LB_174–226_), AB8 (LB_1–46_–LA_50–160_–LB_153–226_), and AB9 (LB_1–71_–LA_83–128_–LB_121–226_), with triple-HA tag at their C-termini. These constructs were cloned into pLenti-Hygro vector via Gibson Assembly (NEB, E2621). The A4GALT (A4)-B4GALT5 (B4) chimera construct A43B352 (A4_1–43_–B4_36–388_) with triple-FLAG tag at the N-termini (with GSGSGSEF as linker) was cloned into pcDNA3.1 vector via Gibson Assembly.

### CRISPR-Cas9 screen

Lentiviral sgRNA plasmid libraries were generated using the human GeCKO-V2 sgRNA library (Addgene, #1000000049). The GeCKO-V2 library is composed of two sub-libraries (sub-library A and sub-library B). Each sub-library contains three unique sgRNA per gene and was independently packed into lentiviral libraries. The titer of lentiviral libraries was calculated as colony-forming units (CFU) per mL. The cell representation was 500, so each sgRNA on average is distributed into 500 cells. The multiplicity of infection (MOI) was 0.2. Polybrene (8 μg/mL, Santa Cruz, sc-134220) was also added to the medium to increase viral transduction efficiency. Cells were cultured in virus-containing medium for 2 d. Infected cells were selected with Puromycin (5 μg/mL, Thermo Scientific, A1113830); 3.3 × 10^7^ (for sub-library A) or 2.9 × 10^7^ (for sub-library B) cells were either saved as Round 0 (R0) samples, or seeded onto four 15-cm culture dishes and exposed to toxins. The survival cells were reseeded and cultured with normal medium without toxins until about 70% confluence. Cells were then subjected to the next round of selection. The remaining cells were harvested. The genomic DNA was extracted using a commercial kit (Qiagen, 13323, Gaithersburg, MD). DNA fragments containing the sgRNA sequences were amplified by PCR using primers lentiGP-1_F (AATGGACTATCATATGCTTACCGTAACTTGAAAGTATTTCG) and lentiGP-3_R (ATGAATACTGCCATTTGTCTCAAGATCTAGTTACGC). NGS was performed by a commercial vendor (Genewiz, Illumina MiSeq, South Plainfield, NJ).

### Mixed and single clone KO cells

The selected sgRNA sequences (S2 Table) were cloned into LentiGuide-Puro vectors (Addgene, #52963). ACHN and 5637 cells that stably express Cas9 were generated using LentiCas9-Blast construct (Addgene, #52962) and were selected using Blasticidin S (10 μg/mL, RPI, B12150.01). HeLa-Cas9 was generously provided by Dr. Abraham Brass (Worcester, MA). These Cas9-expressing cells were transduced with lentiviruses that express selected sgRNAs. The mixed stable cell lines were selected using Puromycin. Single clone of KO cells were generated by diluting the mixed KO cells at about 0.8 cell per well in 48-well plates. The genotype of single-cell clones were determined by amplifying the DNA fragments containing the sgRNA targeting region using the following primers: LAPTM4A_F (CCACAGGTAGTCTCCCACTATTTTATATCTTTGTTACTTC), LAPTM4A_R (GCCTAGGAGTCTCTACCACATCGC), TMEM165_F (TCCGTTGGCCACCATTTTTAGTGCTTCTGAA), TMEM165_R (ATGACTTTGTATTTTGCTAACTTCTACACAGGTG), TM9SF2_F (GAGGAGAGATGTGGTACATAGGACTTGGAG), TM9SF2_R (CTGCCCTTTAAGCCACCGTCTTAAC), followed by ligating the PCR product into T-vectors (Promega, A3600, Madison, WI). The ligation products were transformed into *E*. *coli* (DH5α strain) and plated onto agar plates. Twenty single clone colonies were selected, and their plasmids were extracted and sequenced.

### Cell viability assay

Cells were seeded in 96-well microplates and incubated overnight until about 70% confluence. The medium was replaced with 100 μL toxin-containing medium, with a total of 10 serial dilutions (10-fold). The cells were further incubated for 72 h in toxin-containing medium and then 10 μL MTT (5 mg/mL in PBS) was added to each well and incubated at 37 °C for 4 h. A total of 100 μL solubilization solution (10% SDS in 0.01 M HCl) was then added to each well and incubated overnight. The absorbance of formazan formed was then measured at 580 nm by a microplate reader (BMG Labtech, FLUOstar Omega). A vehicle control without toxins and a blank of PBS were analyzed in parallel. The cytotoxicity curves were analyzed and fitted using Origin software (version 8.5).

### Immunofluorescent staining

Cells were seeded onto glass coverslips in 24-well plates and incubated for 24 h until about 70% confluence. Cells were washed three times with ice-cold PBS, fixed with 4% paraformaldehyde (PFA) for 20 min at room temperature (RT), permeabilized with 0.3% Triton X-100 for 30 min, and blocked with 10% goat serum for 40 min, followed by incubation with primary antibodies (1 h) and fluorescence-labeled secondary antibodies (1 h). Slides were sealed within DAPI-containing mounting medium (SouthernBiotech, 0100–20). We note that for labeling endogenous TM9SF2, cells were fixed in methanol at −20 °C for 10 min, as this antibody did not work on samples fixed by PFA. For the toxin surface binding assay, cells were incubated with toxin-containing medium (4.8 μg/mL Stx1, or 2 ng/mL Ctx-555) on ice for 60 min. Cells were washed, fixed, and subjected to immunostaining without permeabilization. Stx1 polyclonal antibody was used to recognize surface-bound Stx1. Fluorescent images were captured with the Olympus DSU-IX81 Spinning Disk Confocal System. Images were pseudocolored and analyzed using ImageJ.

### Immunoblot analysis

Cells were harvested and washed three times with PBS. The cell pellets were lysed with RIPA buffer (50 mM Tris, pH 7.5, 1% NP40, 150 mM NaCl, 0.5% sodium deoxycholate, 1% SDS, protease inhibitor cocktail). Cell lysates were centrifuged and the protein amounts in supernatants were measured by BCA assay (Thermo Scientific, 23225, Waltham, MA). The supernatants were heat denatured for 5 min, subjected to SDS-PAGE, and transferred onto a nitrocellulose membrane. The membrane was blocked with a TBST buffer (10 mM Tris, pH 7.4, 150 mM NaCl, 0.1% Tween-20) containing 5% skim milk at RT for 1 h. Then, the membrane was incubated with the primary antibodies for 1 h, washed, and incubated with secondary antibodies for 1 h. Signals were detected using the enhanced chemiluminescence method by a Fuji LAS3000 imaging system.

### FACS analysis

For Stx and Ctx binding, cells were incubated with toxin-containing medium (4.8 μg/mL Stx1, or 2 ng/mL Ctx-555) on ice for 60 min, washed three times with ice-cold PBS, and collected. Cell pellets were fixed with 4% PFA for 20 min at RT and blocked with 10% goat serum for 40 min. Stx1 polyclonal antibody was used to recognize surface-bound Stx1 (1 h). Cells were washed and incubated with Alexa488-labeled secondary antibody for 1 h, washed twice, and subjected to single-cell sorting using a Canto II FACS system (BD Biosciences). Cells not treated with toxins were used as controls. For cell surface HS, cells were collected with 1 mM EDTA in PBS and subsequently resuspended in PBS with 1% BSA. Cells were incubated with either the 10E4 monoclonal antibody against HS (1:400, amsbio, 370255, Cambridge MA) or mouse IgM (1:200, abcam, ab18401, Cambridge, MA) for 1 h on ice. Cells were washed twice with PBS and incubated with Alexa488-labeled secondary antibody for 1 h on ice and washed twice, followed by single-cell sorting. Single cells were gated and analyzed using FlowJo software (version 10, FlowJo, Ashland, OR).

### cAMP analysis

Cellular concentrations of cAMP were examined using the Direct Immunoassay Detection Kit (Abcam, ab138880, Cambridge, MA). Briefly, cells were treated with Ctx (50 μg/mL, 4 h), lysed, and the protein amounts were measured. The cell lysates were loaded into anti-cAMP antibody-coated plates. The HRP-linked cAMP was added to compete with the cellular cAMP. The activity of HRP-cAMP conjugate was measured using a microplate reader. A calibration curve of free cAMP was analyzed in parallel. The cAMP concentrations in different cells were normalized by the total protein amount in cell lysates.

### Co-IP assays

HEK293T cells were co-transfected with plasmids encoding FLAG-tagged A4GALT, B4GALT5, or a chimeric protein A43B352, together with HA-tagged LAPTM4A, LAPTM4B, or AB4 at a 1:1 ratio. Cells were harvested 48 h later and cell lysates subjected to co-IP assays using anti-FLAG magnetic beads (Sigma, M8823, St. Louis, MO). Briefly, cells were lysed with RIPA buffer and incubated with anti-FLAG beads overnight at 4 °C. Beads were then washed (0.1% Triton X-100 in PBS), pelleted, and boiled in SDS sample buffer (100 mM Tris, pH 8.0, 4% SDS, 10% glycerol, 0.1% bromophenol blue). After centrifugation, the supernatant (Pull-down) as well as the whole cell lysates (Input) were subjected to immunoblot analysis.

### Glycosphingolipid extraction

Briefly, 3.5×10^6^ cells from each cell line were washed with 0.1% ammonium acetate and suspended in 0.35 mL of deionized water. A mixture of methanol (1.25 mL) and chloroform (0.675 mL) was then added, together with 3,000 ng lipid standards d3-GM2 (d18:1/18:0, Matreya, #2051, State College, PA). The sample was vortexed for 1 min, followed by incubation for 30 min and centrifugation at 2,000*g* for 30 min at RT. Supernatant (the methanol/chloroform fraction) was collected. The remaining cell pellet was resuspended in 250 μL of water, and lipids were extracted again using a methanol/chloroform mixture (2:1 ratio, 1 mL). The supernatant fractions were combined, dried under nitrogen gas, and stored at −20 °C until analysis.

### LC-MS analysis of glycolipids

Lipid extracts were resuspended in 100 μL of 60% methanol and 40% water. For each sample, a total of 5 μL was injected into a Kinetex column (C18, 1.4 μm, 100 Å, 2.1×50 mm; Phenomenex, Torrance, CA) using a UPLC (Waters Corporation, Milford, MA) coupled to a Waters Synapt G2-si quadrupole time-of-flight mass spectrometer fitted with an electrospray ionization source operating in positive ion mode. LC separation was carried out at a flow rate of 0.27 mL/min using mobile phase A: 0.1% formic acid, 5 mM ammonium formate in water, and mobile phase B: 0.1% formic acid, 5 mM ammonium formate in methanol, using the gradient conditions as follows: 0–1 min (60% B), 1–2 min (60%–70% B), 2–40 min (70%–100% B), 40–43 min (100% B), 43–43.1 min (100%–60% B), and 43.1–50 min (60% B). Gangliosides were analyzed in negative ion mode. For each sample, a total of 10 μL was injected onto a Kinetex column and LC-MS system described above. Mobile phase A, 0.1% (v/v) formic acid in water, and mobile phase B, 0.1% (v/v) formic acid, methanol, isopropanol (5/47.5/47.5) at a flow rate of 0.23 mL/min were used for elution using the following gradient conditions: 0–1 min (60% B), 1–12 min (100% B), 12–14 min (100% B), 14–14.1 min (60% B), and 14.1–20 min (60% B). Mass calibration and external lock mass correction were carried out using Glu-1-Fibrinopeptide B. For each lipid detected in positive ion mode (Gb3, LacCer, GlucCer, and Cer), protonated, sodium adduct, and dehydrated ions were detected. Extracted ion chromatograms obtained using a 15-ppm window centered on the theoretical ionic mass of glycosphingolipids were integrated using TargetLynx XS (Waters Corporation); summing of peak areas of the corresponding adducts and further data processing was carried out in Excel (Microsoft, Redmond, WA). Endogenous PC was used as an internal standard to obtain peak area ratios for Gb3, LacCer, GlcCer, and Cer. The corrected peak areas and that of the internal standard d3-GM2 were used to obtain peak area ratios of GM2. Relative standard deviations (RSDs) for the replicate analyses were within 15%–20%.

### Internalization of fluorescently labeled lipids

Cells were precooled on ice and exposed to fluorescently labeled lipids (NBD-Sph, NBD-C6-Cer, and NBD-PS, 5μM) in serum-free medium containing DF-BSA (5 μM) for 40 min at 4 °C. Cells were washed twice with PBS and incubated for the indicated time in serum-free medium containing DF-BSA (5 μM). Cells were then washed three times with ice-cold PBS and fixed with 4% PFA for 20 min at RT. Slides were sealed within DAPI-containing mounting medium.

### Topology analysis

HEK293T cells were transfected with HA-tagged LAPTM4A or EMC1. After fixation in 4% PFA, cells were permeabilized with either Saponin buffer (0.1% Saponin, 0.1% BSA in PBS) for 30 min at RT or Digitonin buffer (5 μg/mL Digitonin, 0.3 M Sucrose, 0.1 M KCl, 2.5 mM MgCl_2_, 1 mM EDTA, 10 mM HEPES, pH 6.9) for 15 min at RT. Cells were then subjected to immunofluorescent staining analysis.

### qRT-PCR assay

The total cellular RNA was extracted by TRIzol (Invitrogen, 15596026, Waltham, MA), quantified, and subjected to a reverse transcription reaction (Applied Biosystems, 4375575, Foster City, CA). The cDNA was quantified and subjected to a qPCR reaction. The reactions were run in triplicate on 96-well plates with an ABI Prism 7700 Sequence Detection System (Applied Biosystems). SYBR Green (Roche, 04913850001) was used to monitor dsDNA synthesis. Glyceraldehyde 3-phosphate dehydrogenase (GAPDH) was used as a housekeeping control to normalize the relative mRNA level. The primers used were as follows: A4GALT_RT_F (GAGACTTCAGACCGGACCAA), A4GALT_RT_R (AAGCCCTTTCATCAGGACCA), LAPTM4A_RT_F (CAAGTGGGTTGGCTGATTCC), LAPTM4A_RT_R (AGGCCAGGAGGTCATCTTTG), GAPDH_RT_F (AGGGCTGCTTTTTAACTCTGGT), and GAPDH_RT_R (CCCCACTTGATTTTGGAGGGA).

## Supporting information

S1 Fig CRISPR-cas9–mediated genome-wide screen for Stx and ricin. (A) Cell viability assays were carried out as described in Fig 1A, except that cells were exposed to Stx2. Error bars indicate mean ± SD, *N* = 3. (B) The sensitivities of the four indicated cell lines to ricin were determined using cell viability assays. HeLa was the most sensitive one among the four cell lines. Error bars indicate mean ± SD, *N* = 3. (C) Schematic diagram of the ricin screen. HeLa cells stably expressing Cas9 were transduced with the human GeCKO-V2 sgRNA library and then selected by increasing concentrations of ricin (0.2, 0.4, 0.8, and 1.5 ng/mL, 48 h). The survival cells were recovered and their sgRNAs were analyzed by NGS. (D) Binding of Stx1 to the cell surface of 5637, ACHN, and HeLa cells was examined by immunostaining using a polyclonal Stx1 antibody. Cells were exposed to Stx1 (4.8 μg/mL) on ice for 60 min, washed, and fixed. Nuclei were labeled with DAPI. ACHN and 5637 cells showed robust binding of Stx1, while binding of Stx1 to HeLa cells was not detectable. Scale bar, 5 μm. Representative images are from one of the three independent experiments. (E) Top genes were enriched in Stx1, Stx2, and ricin screens. For each gene, the number of NGS reads and the number of unique sgRNAs identified from sub-library A and sub-library B were combined. The top 1,000 genes with the highest NGS reads identified in Stx1_R2 (orange circles), Stx2_R2 (purple circles), and Ricin_R4 (green circles) were plotted versus their numbers in R0 (gray circles). The full lists of identified genes were shown in S1 and S2 Data. (F) Gene recovery rates were shown as pie charts for Stx_R0 and Ricin_R0, as compared to the original GeCKO-V2 library. (G) Schematic diagram of Gb3 biosynthesis pathway.
(TIF)

S2 Fig Validating the top-ranked genes using mixed KO cells. (A, B) Mixed stable 5637 KO cells for the indicated genes were generated via the CRISPR-Cas9 approach. For LAPTM4A, two independent KO cell lines using two different sgRNAs were generated (LAPTM4A-KO-Mix and LAPTM4A-KO-II-Mix). We also generated and tested a KO cell line lacking LAPTM4B, a homolog of LAPTM4A. These cells were subjected to cell viability assays for Stx1 (A) or Stx2 (B). The IC_50_ values are listed in S1 Table. Error bars indicate mean ± SD, *N* = 3. (C, D) Mixed LAPTM4A and A4GALT KO ACHN cells were generated via the CRISPR-Cas9 approach and subjected to cell viability assays for Stx1 and Stx2. Both LAPTM4A and A4GALT KO cells showed increased resistance to Stx1 (C) and Stx2 (D). Error bars indicate mean ± SD, *N* = 3. (E) Mixed KO HeLa cells for the selected hits in ricin screen (MGAT2, SLC35C1, GOSR1, ERP44, JTB, TAPT1, NBAS) were generated via the CRISPR-Cas9 approach. These cells were subjected to cell viability assays. The IC_50_ values are listed in S1 Table. Error bars indicate mean ± SD, *N* = 3.
(TIF)

S3 Fig LAPTM4A KO cells lose Stx1 binding. (A) WT and mutant 5637 cells lacking LAPTM4A (LA-KO-10 and LA-KO-12), A4GALT (A4-KO-Mix), or LAPTM4B (LB-KO-Mix) as well as a cell line that expresses a mutated form of LAPTM4A (LA-Mut-9) were exposed to Stx1 (4.8 μg/mL) on ice for 60 min. Cells were washed and cell lysates were subjected to immunoblot analysis detecting bound Stx1 using a polyclonal anti-Stx1 antibody. The A domain of Stx1 (Stx1A) is shown. Actin served as a loading control. Representative images are from one of the three independent experiments. (B) Experiments were carried out as described in panel A, except that cells were analyzed by flow cytometry using Stx1 and Ctx labeled with antibody or fluorescent dyes (Alexa 555), respectively. Cells not exposed to toxins were used as a control (Ctrl). The percentages of cells showing positive toxin binding signals are marked. Representative histograms are from one of the three independent experiments.
(TIF)

S4 Fig Mass spectrometry analysis of glycolipids. (A) The levels of LacCer, GlcCer, and Cer in cells were quantified using mass spectrometry analysis. Ion chromatograms for LacCer, GlcCer, and Cer in indicated cell lines are shown using their respective protonated ion mass centered within 15 ppm for the most abundant fatty acyl chains (16:0 and 24:0 for LacCer and GlcCer, 24:0 for Cer). Quantification was normalized based on using PC as an internal standard. The quantification data are listed in S4 Data. (B) The levels of GM2 in cells were quantified using mass spectrometry analysis, using d3-GM2 as an internal standard. Ion chromatograms for GM2 and d3-GM2 in indicated cell lines are shown using protonated ion mass centered within 15 ppm for the most abundant fatty acyl chains. Quantification was normalized based on d3-GM2. The quantification data are listed in S4 Data.
(TIF)

S5 Fig LAPTM4A is localized in the Golgi in HEK293T and HeLa cells. (A, B) LAPTM4A with a triple C-terminal HA tag (LAPTM4A-HA-C) was expressed in HEK293T (A) and HeLa (B) cells via transient transfection. Cells were subjected to immunostaining detecting the HA tag and six common organelle markers. LAPTM4A-HA-C is colocalized with the Golgi markers Giantin and TGN46. Scale bar, 5 μm. Arrow, colocalization. Representative images are from one of the three independent experiments.
(TIF)

S6 Fig A4GALT is localized in the Golgi and its mRNA levels in LAPTM4A KO cells are similar to WT cells. (A) Endogenous A4GALT in 5637 cells showed a high degree of colocalization with the Golgi marker Giantin. Endogenous A4GALT was detected by immunofluorescent staining using a polyclonal A4GALT antibody. A4GALT KO cells were utilized as a control to confirm the specificity of the A4GALT antibody. (B, C) A4GALT with an N-terminal triple FLAG tag (A4GALT-FLAG-N) was correctly localized in the Golgi when it was expressed in 5637 (B) and HEK293T (C) cells via transient transfection. (D) Ectopic expression of A4GALT-FLAG-N in A4GALT KO cells (A4-KO-Mix) restored binding of Stx1. (E) The mRNA level of A4GALT and LAPTM4A in WT, LA-KO-10, LA-KO-12, A4-KO-Mix, and LB-KO-Mix cells, as well as in LA-KO-10 and LA-KO-12 cells that express LAPTM4A via lentiviral transduction, were determined by qRT-PCR. A4GALT mRNA level is reduced in A4GALT KO cells but remains similar in LAPTM4A KO cells compared to WT cells. Error bars indicate mean ± SD, *N* = 3. *Student’s *t* test, *p* < 0.01. (F) Co-IP experiments were carried out for HEK293T cells co-transfected with HA-tagged LAPTM4A, LAPTM4B, AB4, and FLAG-tagged A4GALT. Samples were analyzed by immunoblot using anti-FLAG and anti-HA antibodies. LAPTM4A, LAPTM4B, and AB4 were co-immunoprecipitated with A4GALT. Scale bar, 5 μm. Arrow, colocalization. Representative images are from one of the three independent experiments.
(TIF)

S7 Fig Determining the topology of LAPTM4A. (A) The protein sequences of LAPTM4A (UniPortKB: Q15012) and LAPTM4B isoform 2 (UniPortKB: Q86VI4-2) were aligned by CLC Sequence Viewer (Version 7.7). The same amino acid residues are labeled in red. LAPTM4A and LAPTM4B have the same domain arrangement with four transmembrane domains (TM1, TM2, TM3, and TM4), two lumen domains (L1 and L2), N-terminal and C-terminal cytosolic domains (C1 and C3), and a short cytosolic linker between the second and third transmembrane domains (C2). The two sgRNA targeting regions are also marked. (B) LAPTM4A with an N-terminal triple HA tag or a C-terminal triple HA tag was expressed in HEK293T cells. Cells were permeabilized with either Saponin, which permeabilizes all the membranes, or Digitonin, which only permeabilizes the plasma membrane. The accessibility of the HA tag was assessed by immunostaining using an anti-HA antibody. Both HA-N and HA-C were detected with either Saponin or Digitonin, suggesting that both the C- and N-termini of LAPTM4A are localized in the cytosol. EMC1 with an N-terminal HA tag or a C-terminal HA tag was analyzed in parallel as a control, which showed no signaling for the N-terminal tagged version under Digitonin treatment. Scale bar, 10 μm. Representative images are from one of the three independent experiments. (C) Schematic drawings depicting the topology of LAPTM4A and EMC1.
(TIF)

S8 Fig The second luminal domain of LAPTM4A is critical for biosynthesis of Gb3. (A) Indicated chimeric proteins between LAPTM4A and LAPTM4B were expressed in LAPTM4A KO cells. Binding of Stx1 to these cells was examined by flow cytometry. The percentages of cells showing positive toxin-binding signals are marked. Representative histograms are from one of the three independent experiments. (B) Experiments were carried out as described in panel A, except that binding of Stx1 was assessed by immunoblot analysis of cell lysates. All chimeric proteins contain a triple HA tag on their C-terminal, and their expression was confirmed using a HA antibody. Stx1 was detected using a polyclonal Stx1 antibody, and the A domain was shown (Stx1A). Actin served as a loading control. Representative images were from one of the three independent experiments.
(TIF)

S9 Fig TMEM165 is localized in the Golgi and its deficiency reduces Stx1 binding. (A) Experiments were carried out as described in Fig 4F, except that binding of Stx1 was assessed by immunoblot analysis of cell lysates. Representative images are from one of the three independent experiments. (B) Experiments were carried out as described in Fig 5B, except that cells were also exposed to fluorescently labeled CtxB and binding of Stx1 and CtxB were assessed by flow cytometry. The percentages of cells showing positive toxin binding signals are marked. Representative histograms are from one of the two independent experiments. (C, D) HEK293T (C) and HeLa (D) cells were transfected with N-terminal HA-tagged TMEM165 via transient transfection, and cells were subjected to immunostaining analysis. The HA signals were largely colocalized with the Golgi markers Giantin and TGN46. Scale bar, 5 μm. Arrow, colocalization. Representative images are from one of the three independent experiments. (E) Cells from cell line 5637 were transfected with N-terminal HA-tagged TMEM165 via transient transfection. The expression of TMEM165 was confirmed by immunoblot analysis of cell lysates using an HA antibody. Actin served as a loading control. Representative images were from one of the two independent experiments.
(TIF)

S10 Fig TM9SF2 KO cells lose binding of Stx and Ctx. (A) WT, TM9SF2 KO cells (SF2-KO-8 and SF2-KO-9), and a cell line that still expresses WT TM9SF2 (SF2-WT-5) were exposed to Stx1. Surface-bound Stx1 was detected by immunoblot analysis of cell lysates. Representative images are from one of the three independent experiments. (B) Experiments were carried out as described in A, except that cells were also exposed to fluorescently labeled CtxB, and binding of Stx1 and CtxB were analyzed by flow cytometry. Representative histograms are from one of the two independent experiments.
(TIF)

S11 Fig TM9SF2 is localized in the Golgi, and TM9SF2 KO cells showed dysfunction in endosomal trafficking. (A) The specificity of the TM9SF2 polyclonal antibody is validated, as it showed no signal on TM9SF2 KO 5637 cells and HeLa cells (mixed KO cells were generated via CRISPR-Cas9 approach). (B, C) The endogenous TM9SF2 in HEK293T (B) and HeLa (C) cells was colocalized with the Golgi marker Giantin and TGN46. Scale bar, 5 μm. Arrow, colocalization. Representative images were from one of the three independent experiments. (D, E) TM9SF2 KO cells showed dysfunction in trafficking of internalized Cer and PS. WT, SF2-WT-5, SF2-KO-8, and SF2-KO-9 cells were loaded with NBD-labeled six-carbon Cer (NBD-C6-Cer, D) or PS (NBD-PS, E) on ice and then incubated at 37 °C for the indicated time. For WT and SF2-WT-5 cells, the NBD-C6-Cer and NBD-PS fluorescent signals showed as punctate dots at 10 min or 0.5 h, respectively. The fluorescent signals were then disbursed throughout the cells. For SF2-KO-8 and SF2-KO-9 cells, the NBD-PS fluorescent signals remain as punctate dots after 5 h, while the NBD-C6-Cer fluorescent signals remain as punctate dots even 24 h later. Scale bar, 5 μm. Representative images are from one of the two independent experiments. (F) Mixed TM9SF2 KO HeLa cells also showed dysfunction in trafficking of NBD-labeled Sph, Cer, and PS similar to TM9SF2 KO 5637 cells. Scale bar, 5 μm. Representative images are from one of the two independent experiments.
(TIF)

S12 Fig Quantification of the colocalization of LAPTM4A, A4GALT, TMEM165, and TM9SF2 with organelle markers. The colocalization of C-terminal HA-tagged LAPTM4A (Fig 3A and S5A and S5B Fig), N-terminal FLAG-tagged A4GALT (S6B and S6C Fig), C-terminal HA-tagged TMEM165 (Fig 5A and S9C and S9D Fig), and endogenous TM9SF2 (Fig 7A and S11B and S11C Fig) with different organelle markers was analyzed using ImageJ. The Mander’s coefficient obtained was utilized as the colocalization score. Six to ten individual cells were analyzed and shown as box charts. Error bars indicate mean ± SD.
(TIF)

S1 Table The IC_50_ of Stx1, Stx2, and ricin against the indicated cell lines. (DOCX)

S2 Table The sgRNA sequence used to generate KO cell lines. (DOCX)

S3 Table Genotypes of KO single clones and their relative sensitivities to toxins (compared with WT cells). (DOCX)

S1 Data Full list of Stx screens results. (XLSX)

S2 Data Full list of ricin screens results. (XLSX)

S3 Data Genotyping of single clones. (XLSX)

S4 Data Mass spectrometry-based lipidomics data. (XLSX)

S5 Data All numerical data. (XLSX)

## Abbreviations

A4GALT: *α*-1,4-galactosyltransferase
A-Dtx: anthrax-diphtheria chimeric toxin
ARCN1: coatomer subunit delta
B4GALT5: *β*-1,4-galactosyltransferase 5
C1GALT1: core 1 β3galactosyltransferase
CDG: congenital disorders of glycosylation
Cer: ceramide
co-IP: co-immunoprecipitation
COPI: the coat protein complex I
CRISPR: Clustered Regularly Interspaced Short Palindromic Repeats
Ctx: cholera toxin
CtxB: receptor-binding domain of Ctx
DTA: enzymatic domain of diphtheria toxin
EHEC: enterohemorrhagic *Escherichia coli*
EMC1: ER membrane protein complex subunit 1
ER: endoplasmic reticulum
ERAD: ER-associated protein degradation
ERP44: ER resident protein 44
GARP: Golgi-associated retrograde protein
Gb3: globotriaosylceramide
GFP: green fluorescent protein
GlcCer: glucosylceramide
GOSR1: Golgi SNAP receptor complex member 1
GT: glycosyltransferase
HS: heparan sulfate
HUS: hemolytic uremic syndrome
JTB: , jumping translocation breakpoint protein
KD: knockdown
KO: knockout
LacCer: lactosylceramide
LAPTM4A: lysosomal-associated protein transmembrane 4 alpha
LAPTM4B: lysosomal-associated protein transmembrane 4 beta
LFn: N-terminal part of anthrax toxin lethal factor
NBAS: neuroblastoma-amplified sequence
NBD: nitrobenzoxadiazole
NGS: next-generation sequencing
NT: nucleotide transporter
PC: phosphatidylcholine
PS: phosphatidylserine
sgRNA: single guide ribonucleic acid
siRNA: small interfering ribonucleic acid
Sph: sphingosine
SPPL3: signal peptide peptidase-like 3
SPTSSA: serine palmitoyltransferase small subunit A
Stxs: Shiga and Shiga-like toxins
TM9SF2: Transmembrane 9 superfamily member 2
TMEM165: Transmembrane protein 165
UBE2G2: ubiquitin conjugating enzyme E2 G2
UGCG: ceramide glucosyltransferase
UGP2: UDP-Glucose Pyrophosphorylase 2
UPLC: ultra-pressure liquid chromatograph
WT: wild-type
