# Supplementary figures and images for: Genome-wide CRISPR screens for Shiga toxins and ricin reveal Golgi proteins critical for glycosylation
Source: PLoS Biol. 2018 Nov 27;16(11):e2006951. doi: 10.1371/journal.pbio.2006951 (PMC6258472; doi:10.1371/journal.pbio.2006951)

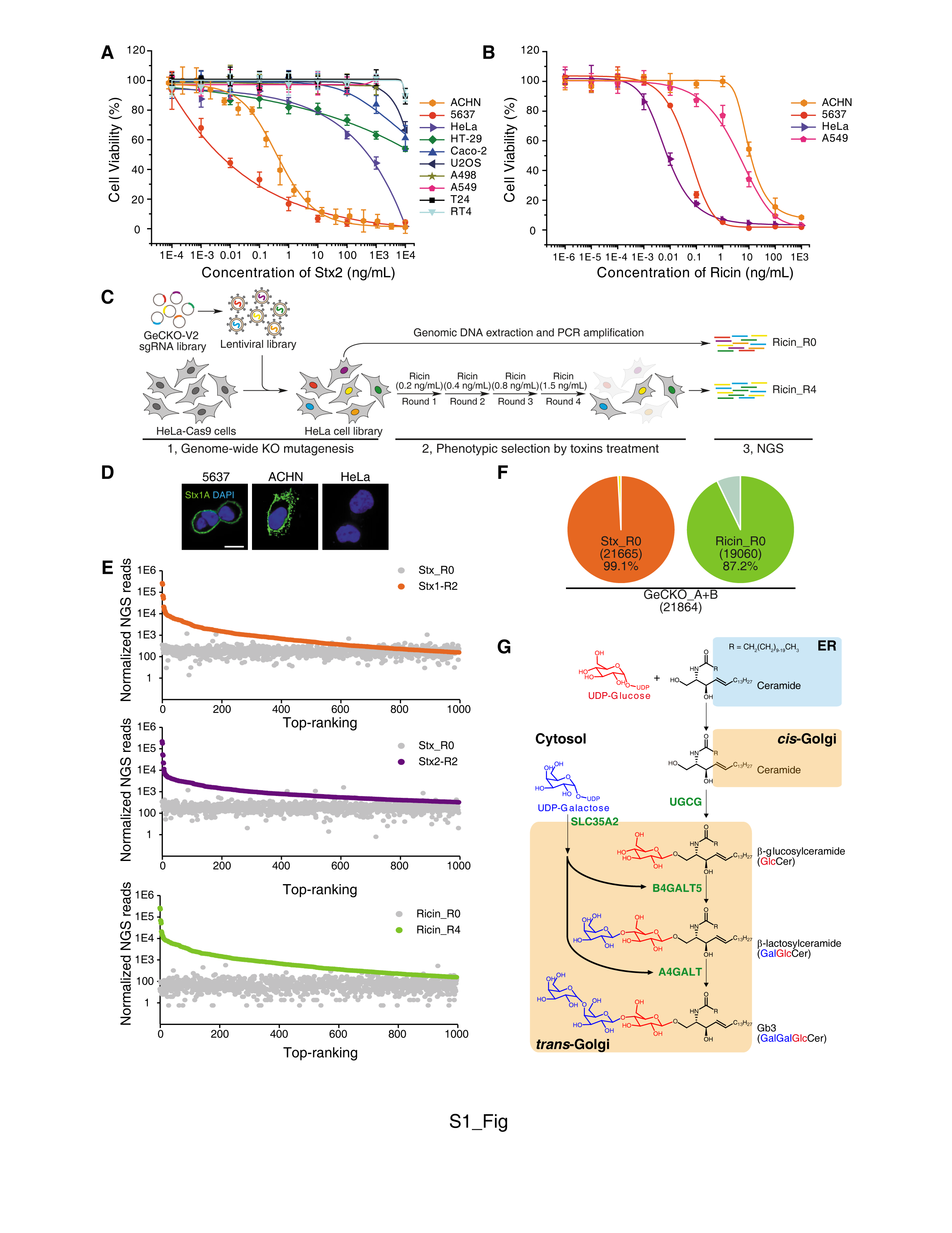

Supplement: S1 Fig — (A) Cell viability assays were carried out as described in Fig 1A, except that cells were exposed to Stx2. Error bars indicate mean ± SD, N = 3. (B) The sensitivities of the four indicated cell lines to ricin were determined using cell viability assays. HeLa was the most sensitive one among the four cell lines. Error bars indicate mean ± SD, N = 3. (C) Schematic diagram of the ricin screen. HeLa cells stably expressing Cas9 were transduced with the human GeCKO-V2 sgRNA library and then selected by increasing concentrations of ricin (0.2, 0.4, 0.8, and 1.5 ng/mL, 48 h). The survival cells were recovered and their sgRNAs were analyzed by NGS. (D) Binding of Stx1 to the cell surface of 5637, ACHN, and HeLa cells was examined by immunostaining using a polyclonal Stx1 antibody. Cells were exposed to Stx1 (4.8 μg/mL) on ice for 60 min, washed, and fixed. Nuclei were labeled with DAPI. ACHN and 5637 cells showed robust binding of Stx1, while binding of Stx1 to HeLa cells was not detectable. Scale bar, 5 μm. Representative images are from one of the three independent experiments. (E) Top genes were enriched in Stx1, Stx2, and ricin screens. For each gene, the number of NGS reads and the number of unique sgRNAs identified from sub-library A and sub-library B were combined. The top 1,000 genes with the highest NGS reads identified in Stx1_R2 (orange circles), Stx2_R2 (purple circles), and Ricin_R4 (green circles) were plotted versus their numbers in R0 (gray circles). The full lists of identified genes were shown in S1 and S2 Data. (F) Gene recovery rates were shown as pie charts for Stx_R0 and Ricin_R0, as compared to the original GeCKO-V2 library. (G) Schematic diagram of Gb3 biosynthesis pathway. (TIF) [file pbio.2006951.s001.tif]

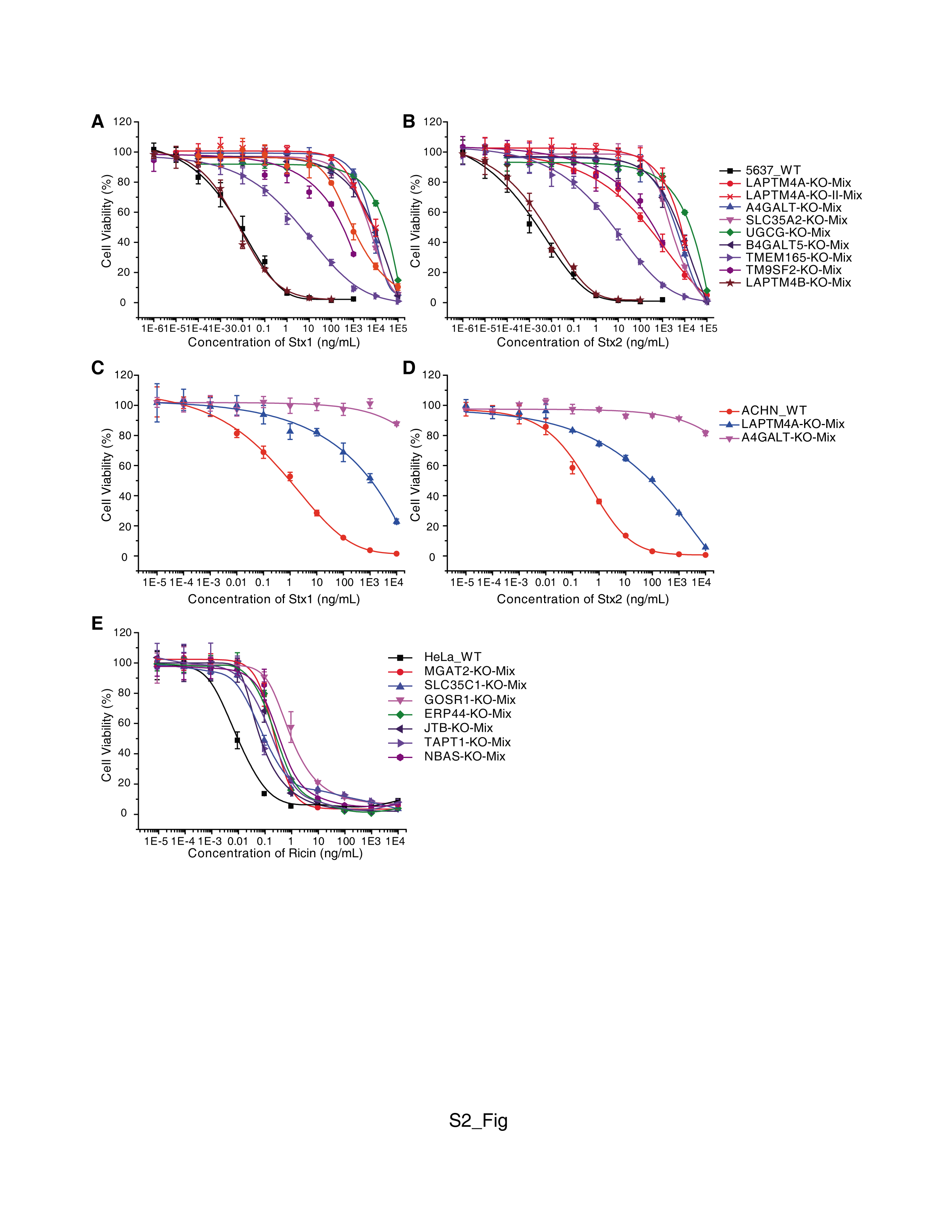

Supplement: S2 Fig — (A, B) Mixed stable 5637 KO cells for the indicated genes were generated via the CRISPR-Cas9 approach. For LAPTM4A, two independent KO cell lines using two different sgRNAs were generated (LAPTM4A-KO-Mix and LAPTM4A-KO-II-Mix). We also generated and tested a KO cell line lacking LAPTM4B, a homolog of LAPTM4A. These cells were subjected to cell viability assays for Stx1 (A) or Stx2 (B). The IC50 values are listed in S1 Table. Error bars indicate mean ± SD, N = 3. (C, D) Mixed LAPTM4A and A4GALT KO ACHN cells were generated via the CRISPR-Cas9 approach and subjected to cell viability assays for Stx1 and Stx2. Both LAPTM4A and A4GALT KO cells showed increased resistance to Stx1 (C) and Stx2 (D). Error bars indicate mean ± SD, N = 3. (E) Mixed KO HeLa cells for the selected hits in ricin screen (MGAT2, SLC35C1, GOSR1, ERP44, JTB, TAPT1, NBAS) were generated via the CRISPR-Cas9 approach. These cells were subjected to cell viability assays. The IC50 values are listed in S1 Table. Error bars indicate mean ± SD, N = 3. (TIF) [file pbio.2006951.s002.tif]

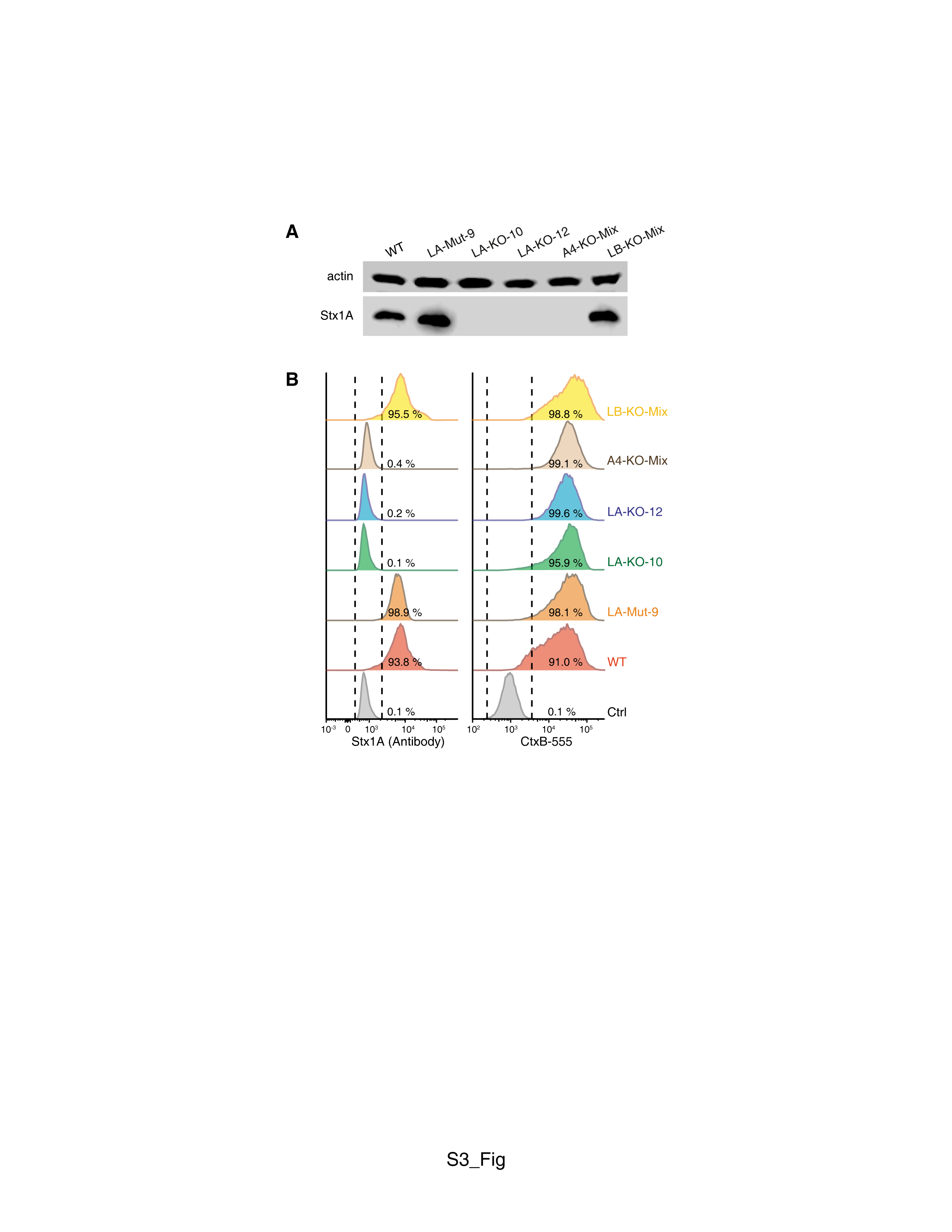

Supplement: S3 Fig — (A) WT and mutant 5637 cells lacking LAPTM4A (LA-KO-10 and LA-KO-12), A4GALT (A4-KO-Mix), or LAPTM4B (LB-KO-Mix) as well as a cell line that expresses a mutated form of LAPTM4A (LA-Mut-9) were exposed to Stx1 (4.8 μg/mL) on ice for 60 min. Cells were washed and cell lysates were subjected to immunoblot analysis detecting bound Stx1 using a polyclonal anti-Stx1 antibody. The A domain of Stx1 (Stx1A) is shown. Actin served as a loading control. Representative images are from one of the three independent experiments. (B) Experiments were carried out as described in panel A, except that cells were analyzed by flow cytometry using Stx1 and Ctx labeled with antibody or fluorescent dyes (Alexa 555), respectively. Cells not exposed to toxins were used as a control (Ctrl). The percentages of cells showing positive toxin binding signals are marked. Representative histograms are from one of the three independent experiments. (TIF) [file pbio.2006951.s003.tif]

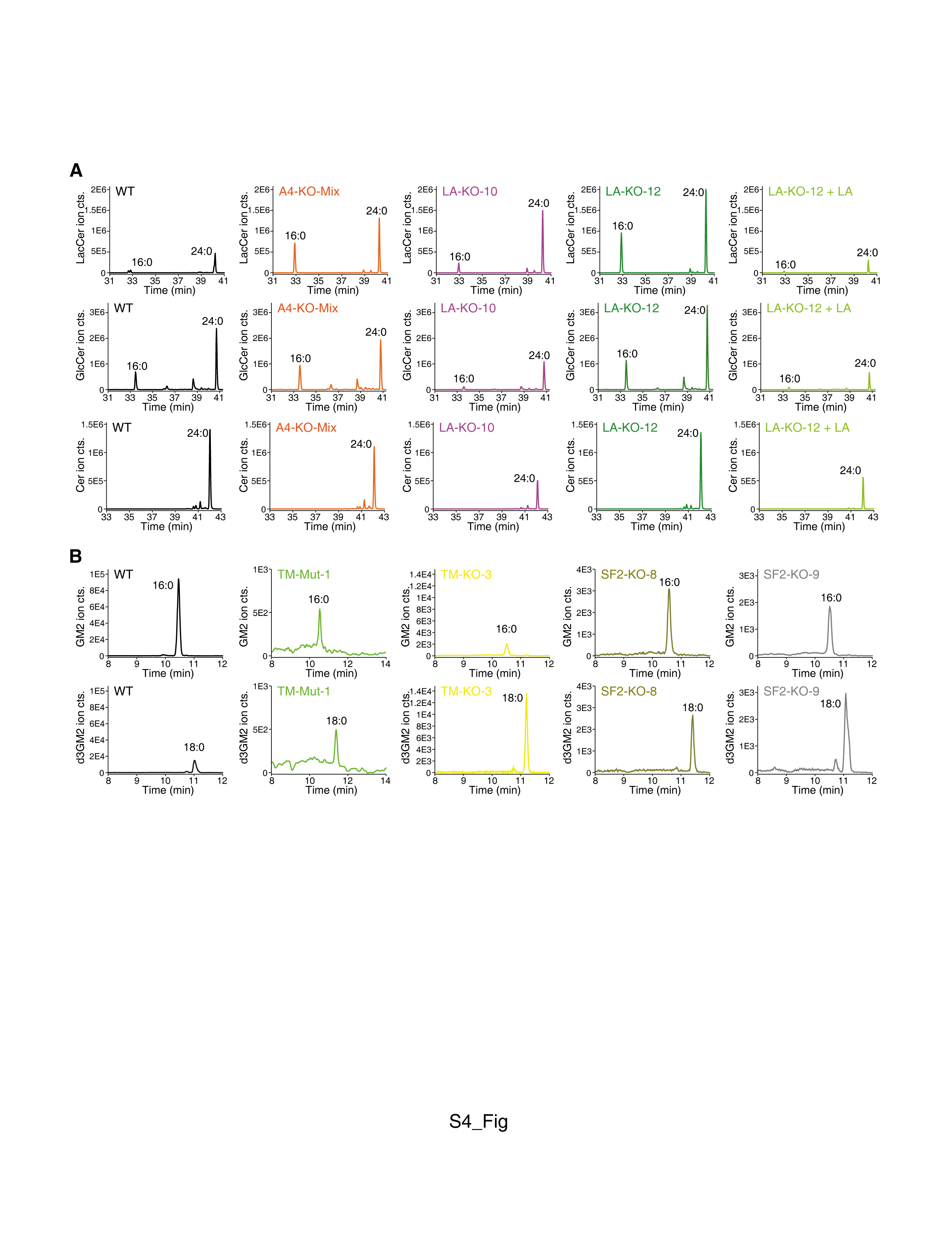

Supplement: S4 Fig — (A) The levels of LacCer, GlcCer, and Cer in cells were quantified using mass spectrometry analysis. Ion chromatograms for LacCer, GlcCer, and Cer in indicated cell lines are shown using their respective protonated ion mass centered within 15 ppm for the most abundant fatty acyl chains (16:0 and 24:0 for LacCer and GlcCer, 24:0 for Cer). Quantification was normalized based on using PC as an internal standard. The quantification data are listed in S4 Data. (B) The levels of GM2 in cells were quantified using mass spectrometry analysis, using d3-GM2 as an internal standard. Ion chromatograms for GM2 and d3-GM2 in indicated cell lines are shown using protonated ion mass centered within 15 ppm for the most abundant fatty acyl chains. Quantification was normalized based on d3-GM2. The quantification data are listed in S4 Data. (TIF) [file pbio.2006951.s004.tif]

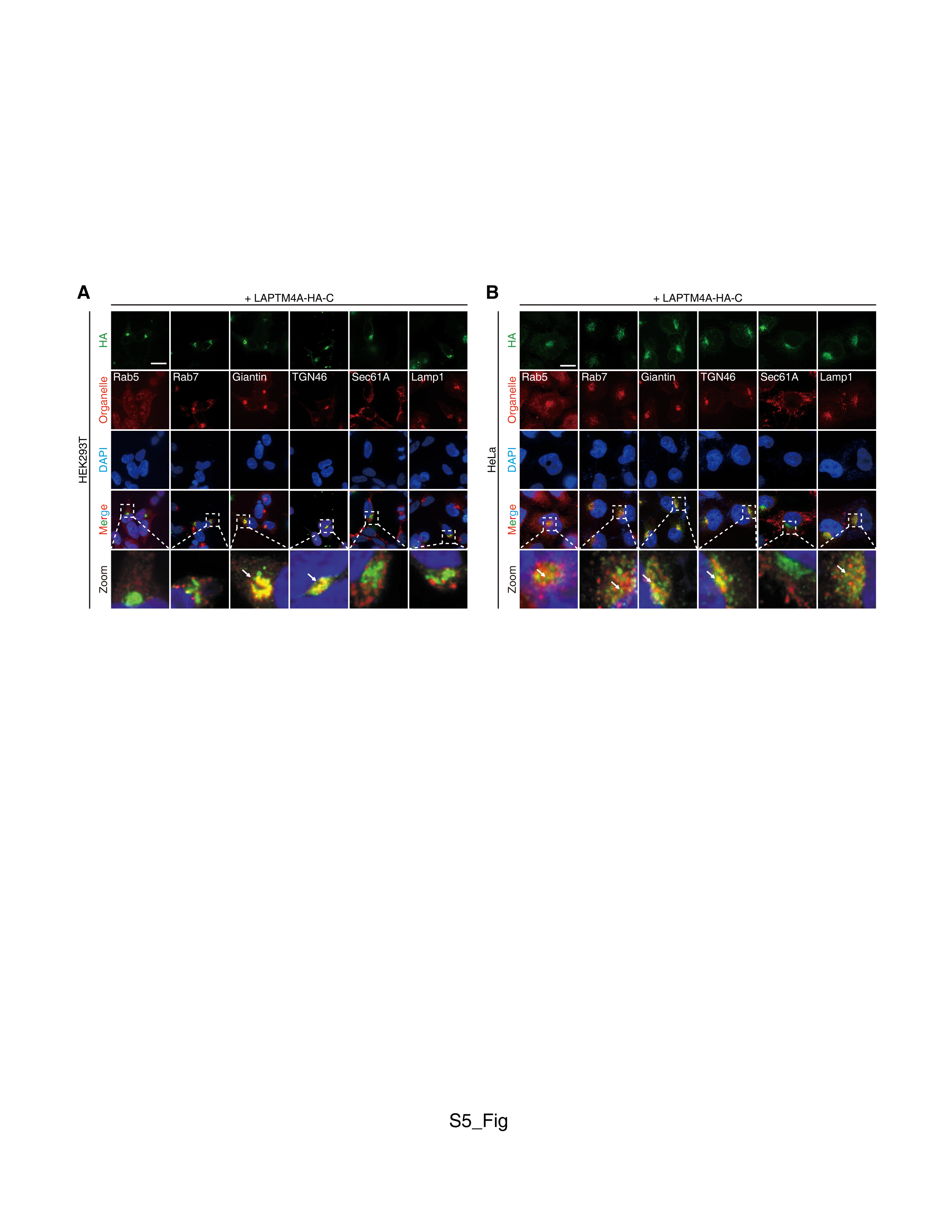

Supplement: S5 Fig — (A, B) LAPTM4A with a triple C-terminal HA tag (LAPTM4A-HA-C) was expressed in HEK293T (A) and HeLa (B) cells via transient transfection. Cells were subjected to immunostaining detecting the HA tag and six common organelle markers. LAPTM4A-HA-C is colocalized with the Golgi markers Giantin and TGN46. Scale bar, 5 μm. Arrow, colocalization. Representative images are from one of the three independent experiments. (TIF) [file pbio.2006951.s005.tif]

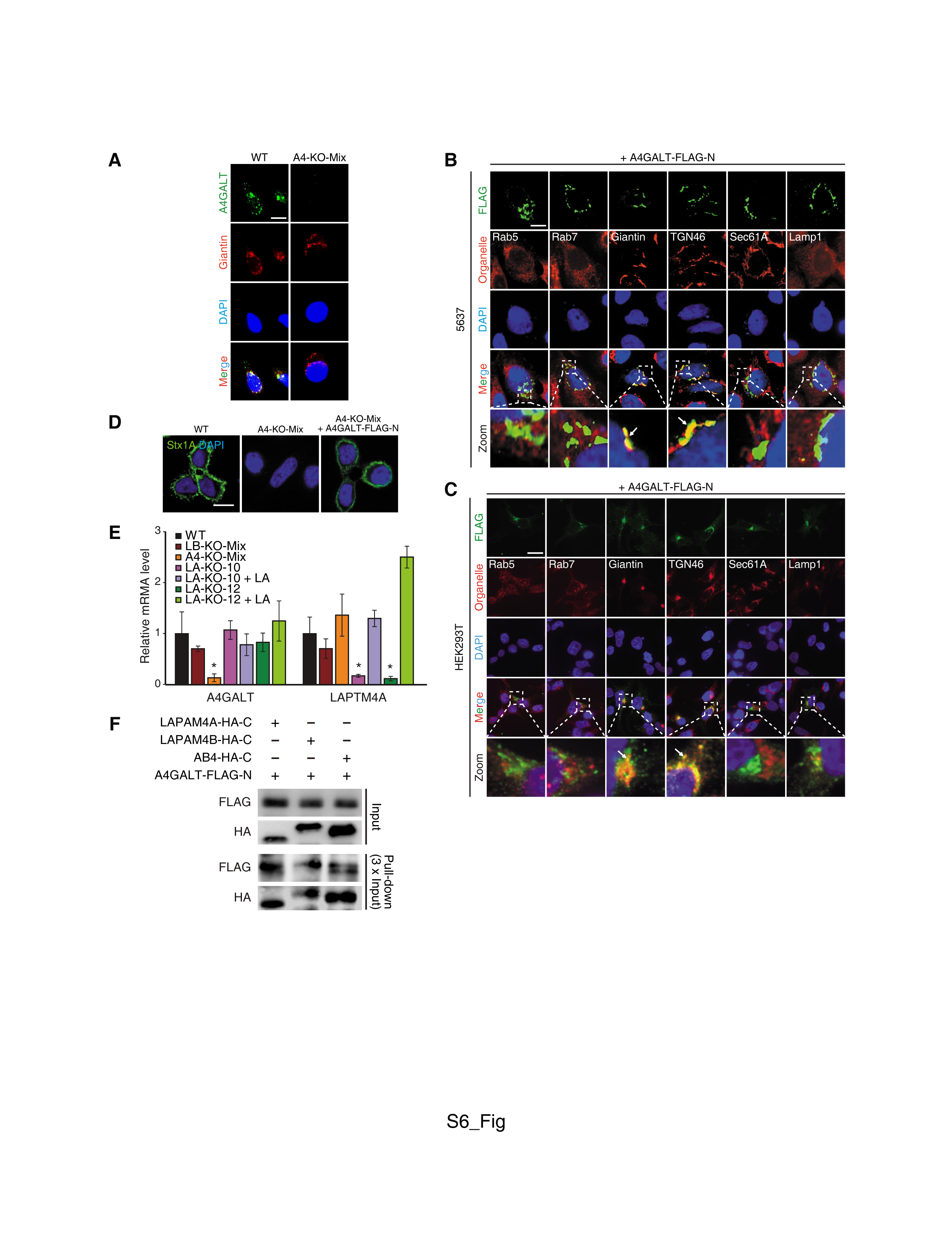

Supplement: S6 Fig — (A) Endogenous A4GALT in 5637 cells showed a high degree of colocalization with the Golgi marker Giantin. Endogenous A4GALT was detected by immunofluorescent staining using a polyclonal A4GALT antibody. A4GALT KO cells were utilized as a control to confirm the specificity of the A4GALT antibody. (B, C) A4GALT with an N-terminal triple FLAG tag (A4GALT-FLAG-N) was correctly localized in the Golgi when it was expressed in 5637 (B) and HEK293T (C) cells via transient transfection. (D) Ectopic expression of A4GALT-FLAG-N in A4GALT KO cells (A4-KO-Mix) restored binding of Stx1. (E) The mRNA level of A4GALT and LAPTM4A in WT, LA-KO-10, LA-KO-12, A4-KO-Mix, and LB-KO-Mix cells, as well as in LA-KO-10 and LA-KO-12 cells that express LAPTM4A via lentiviral transduction, were determined by qRT-PCR. A4GALT mRNA level is reduced in A4GALT KO cells but remains similar in LAPTM4A KO cells compared to WT cells. Error bars indicate mean ± SD, N = 3. *Student’s t test, p < 0.01. (F) Co-IP experiments were carried out for HEK293T cells co-transfected with HA-tagged LAPTM4A, LAPTM4B, AB4, and FLAG-tagged A4GALT. Samples were analyzed by immunoblot using anti-FLAG and anti-HA antibodies. LAPTM4A, LAPTM4B, and AB4 were co-immunoprecipitated with A4GALT. Scale bar, 5 μm. Arrow, colocalization. Representative images are from one of the three independent experiments. (TIF) [file pbio.2006951.s006.tif]

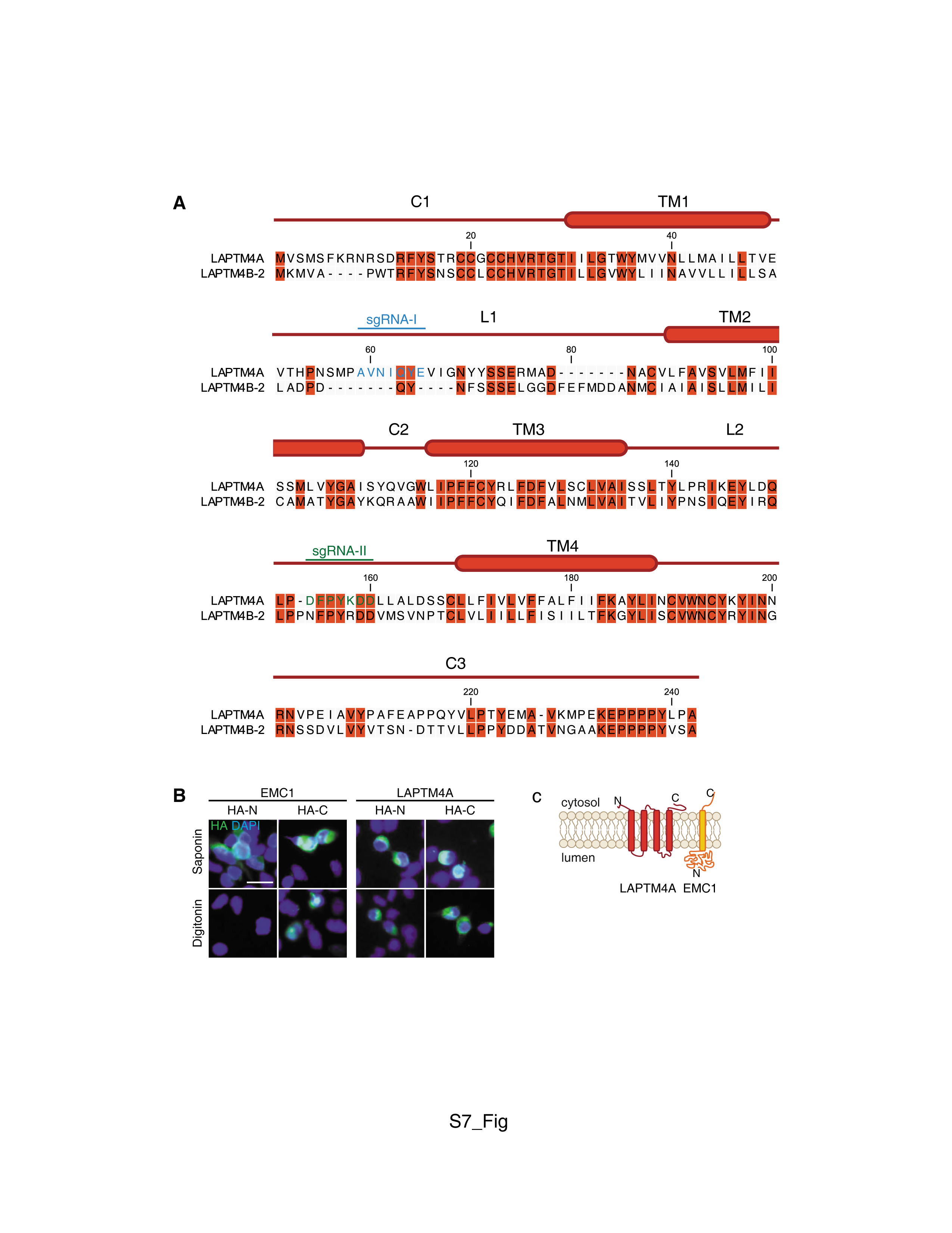

Supplement: S7 Fig — (A) The protein sequences of LAPTM4A (UniPortKB: Q15012) and LAPTM4B isoform 2 (UniPortKB: Q86VI4-2) were aligned by CLC Sequence Viewer (Version 7.7). The same amino acid residues are labeled in red. LAPTM4A and LAPTM4B have the same domain arrangement with four transmembrane domains (TM1, TM2, TM3, and TM4), two lumen domains (L1 and L2), N-terminal and C-terminal cytosolic domains (C1 and C3), and a short cytosolic linker between the second and third transmembrane domains (C2). The two sgRNA targeting regions are also marked. (B) LAPTM4A with an N-terminal triple HA tag or a C-terminal triple HA tag was expressed in HEK293T cells. Cells were permeabilized with either Saponin, which permeabilizes all the membranes, or Digitonin, which only permeabilizes the plasma membrane. The accessibility of the HA tag was assessed by immunostaining using an anti-HA antibody. Both HA-N and HA-C were detected with either Saponin or Digitonin, suggesting that both the C- and N-termini of LAPTM4A are localized in the cytosol. EMC1 with an N-terminal HA tag or a C-terminal HA tag was analyzed in parallel as a control, which showed no signaling for the N-terminal tagged version under Digitonin treatment. Scale bar, 10 μm. Representative images are from one of the three independent experiments. (C) Schematic drawings depicting the topology of LAPTM4A and EMC1. (TIF) [file pbio.2006951.s007.tif]

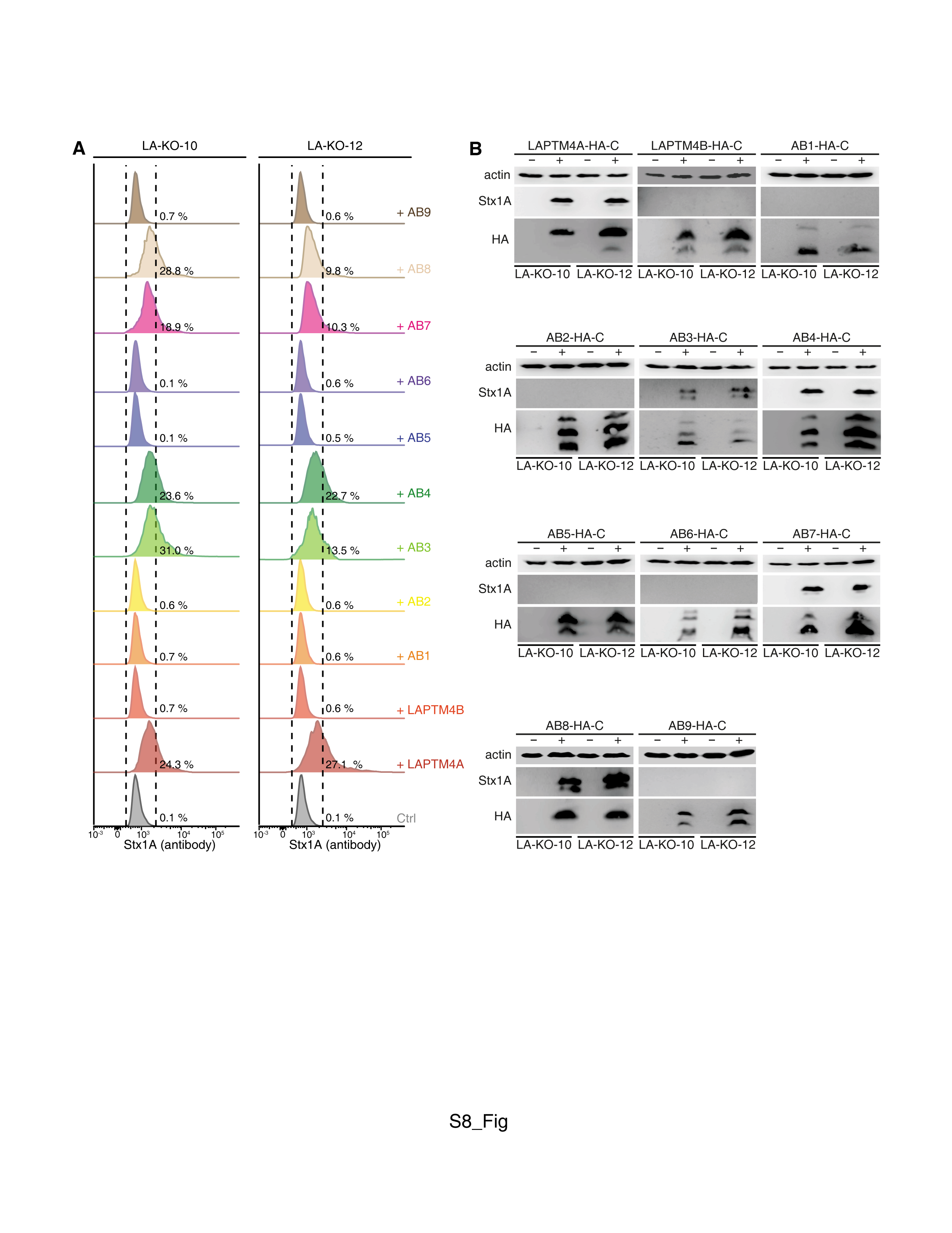

Supplement: S8 Fig — (A) Indicated chimeric proteins between LAPTM4A and LAPTM4B were expressed in LAPTM4A KO cells. Binding of Stx1 to these cells was examined by flow cytometry. The percentages of cells showing positive toxin-binding signals are marked. Representative histograms are from one of the three independent experiments. (B) Experiments were carried out as described in panel A, except that binding of Stx1 was assessed by immunoblot analysis of cell lysates. All chimeric proteins contain a triple HA tag on their C-terminal, and their expression was confirmed using a HA antibody. Stx1 was detected using a polyclonal Stx1 antibody, and the A domain was shown (Stx1A). Actin served as a loading control. Representative images were from one of the three independent experiments. (TIF) [file pbio.2006951.s008.tif]

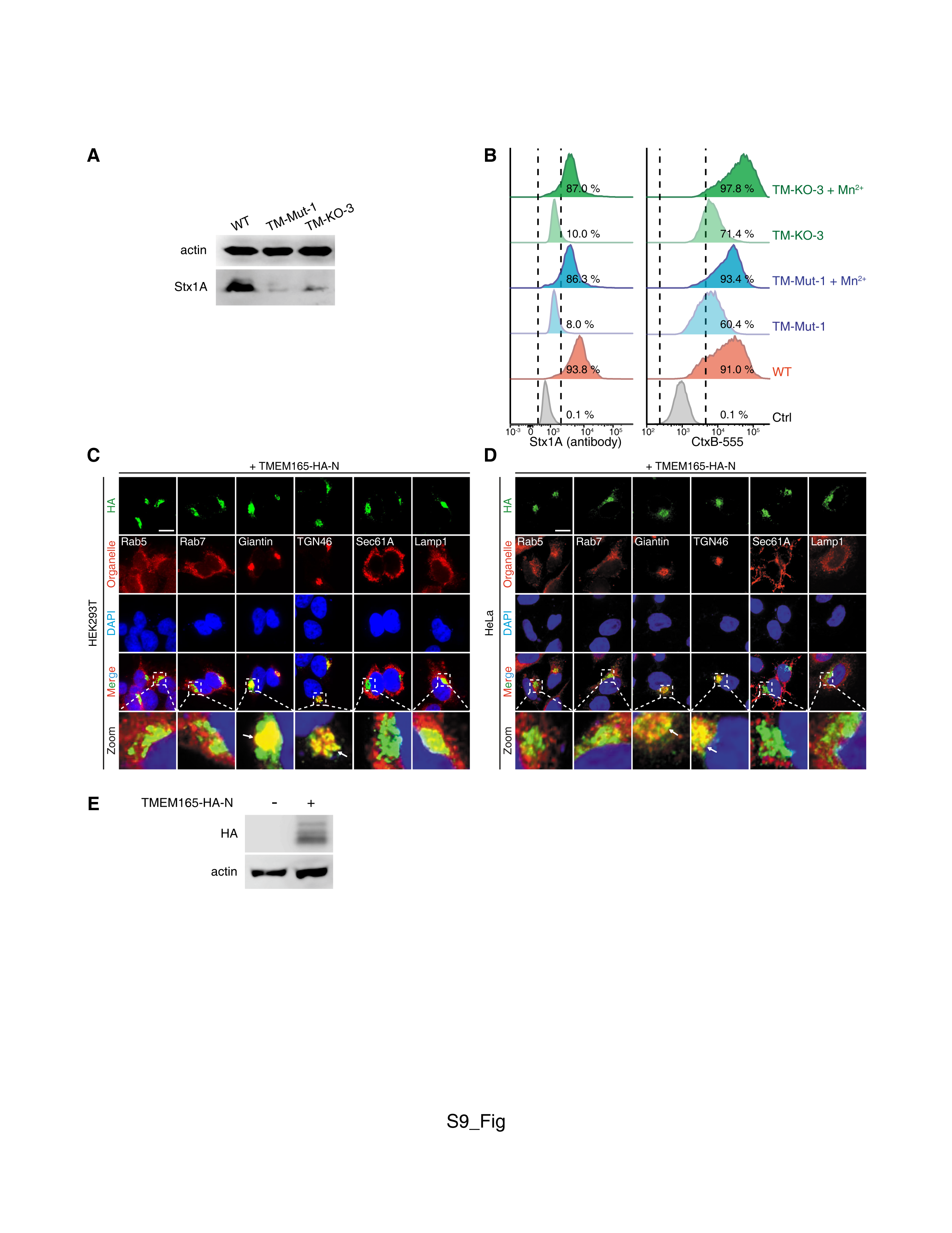

Supplement: S9 Fig — (A) Experiments were carried out as described in Fig 4F, except that binding of Stx1 was assessed by immunoblot analysis of cell lysates. Representative images are from one of the three independent experiments. (B) Experiments were carried out as described in Fig 5B, except that cells were also exposed to fluorescently labeled CtxB and binding of Stx1 and CtxB were assessed by flow cytometry. The percentages of cells showing positive toxin binding signals are marked. Representative histograms are from one of the two independent experiments. (C, D) HEK293T (C) and HeLa (D) cells were transfected with N-terminal HA-tagged TMEM165 via transient transfection, and cells were subjected to immunostaining analysis. The HA signals were largely colocalized with the Golgi markers Giantin and TGN46. Scale bar, 5 μm. Arrow, colocalization. Representative images are from one of the three independent experiments. (E) Cells from cell line 5637 were transfected with N-terminal HA-tagged TMEM165 via transient transfection. The expression of TMEM165 was confirmed by immunoblot analysis of cell lysates using an HA antibody. Actin served as a loading control. Representative images were from one of the two independent experiments. (TIF) [file pbio.2006951.s009.tif]

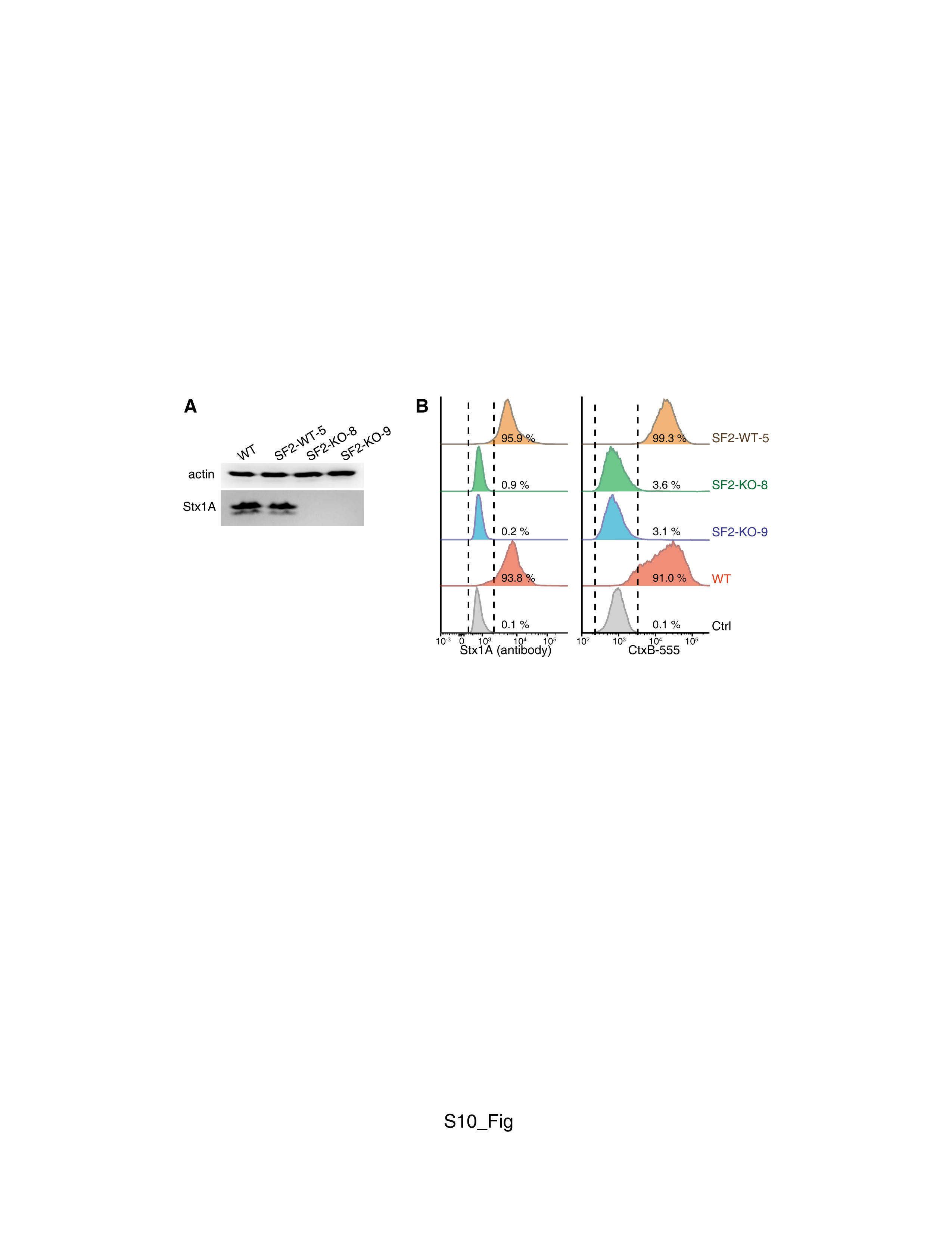

Supplement: S10 Fig — (A) WT, TM9SF2 KO cells (SF2-KO-8 and SF2-KO-9), and a cell line that still expresses WT TM9SF2 (SF2-WT-5) were exposed to Stx1. Surface-bound Stx1 was detected by immunoblot analysis of cell lysates. Representative images are from one of the three independent experiments. (B) Experiments were carried out as described in A, except that cells were also exposed to fluorescently labeled CtxB, and binding of Stx1 and CtxB were analyzed by flow cytometry. Representative histograms are from one of the two independent experiments. (TIF) [file pbio.2006951.s010.tif]

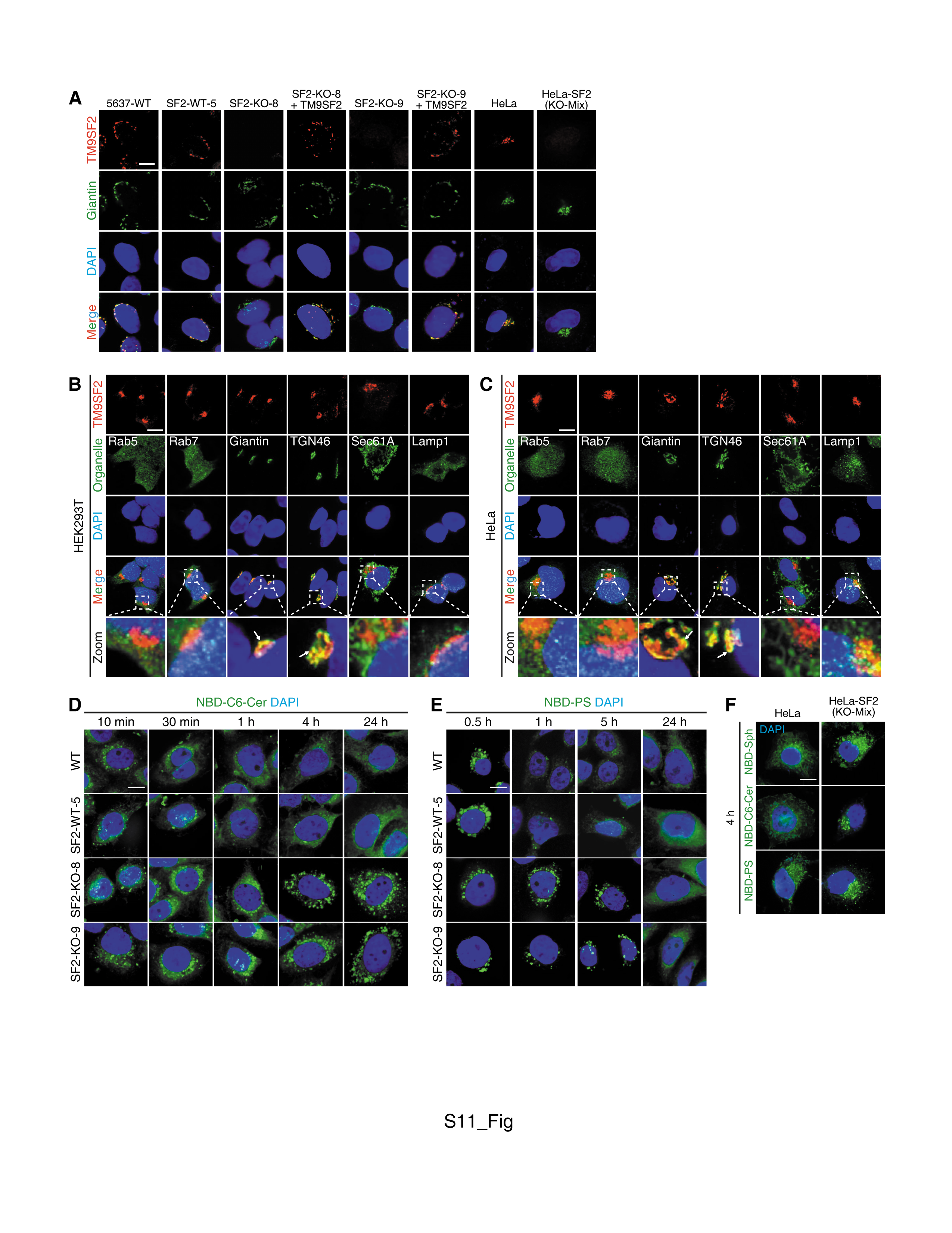

Supplement: S11 Fig — (A) The specificity of the TM9SF2 polyclonal antibody is validated, as it showed no signal on TM9SF2 KO 5637 cells and HeLa cells (mixed KO cells were generated via CRISPR-Cas9 approach). (B, C) The endogenous TM9SF2 in HEK293T (B) and HeLa (C) cells was colocalized with the Golgi marker Giantin and TGN46. Scale bar, 5 μm. Arrow, colocalization. Representative images were from one of the three independent experiments. (D, E) TM9SF2 KO cells showed dysfunction in trafficking of internalized Cer and PS. WT, SF2-WT-5, SF2-KO-8, and SF2-KO-9 cells were loaded with NBD-labeled six-carbon Cer (NBD-C6-Cer, D) or PS (NBD-PS, E) on ice and then incubated at 37 °C for the indicated time. For WT and SF2-WT-5 cells, the NBD-C6-Cer and NBD-PS fluorescent signals showed as punctate dots at 10 min or 0.5 h, respectively. The fluorescent signals were then disbursed throughout the cells. For SF2-KO-8 and SF2-KO-9 cells, the NBD-PS fluorescent signals remain as punctate dots after 5 h, while the NBD-C6-Cer fluorescent signals remain as punctate dots even 24 h later. Scale bar, 5 μm. Representative images are from one of the two independent experiments. (F) Mixed TM9SF2 KO HeLa cells also showed dysfunction in trafficking of NBD-labeled Sph, Cer, and PS similar to TM9SF2 KO 5637 cells. Scale bar, 5 μm. Representative images are from one of the two independent experiments. (TIF) [file pbio.2006951.s011.tif]

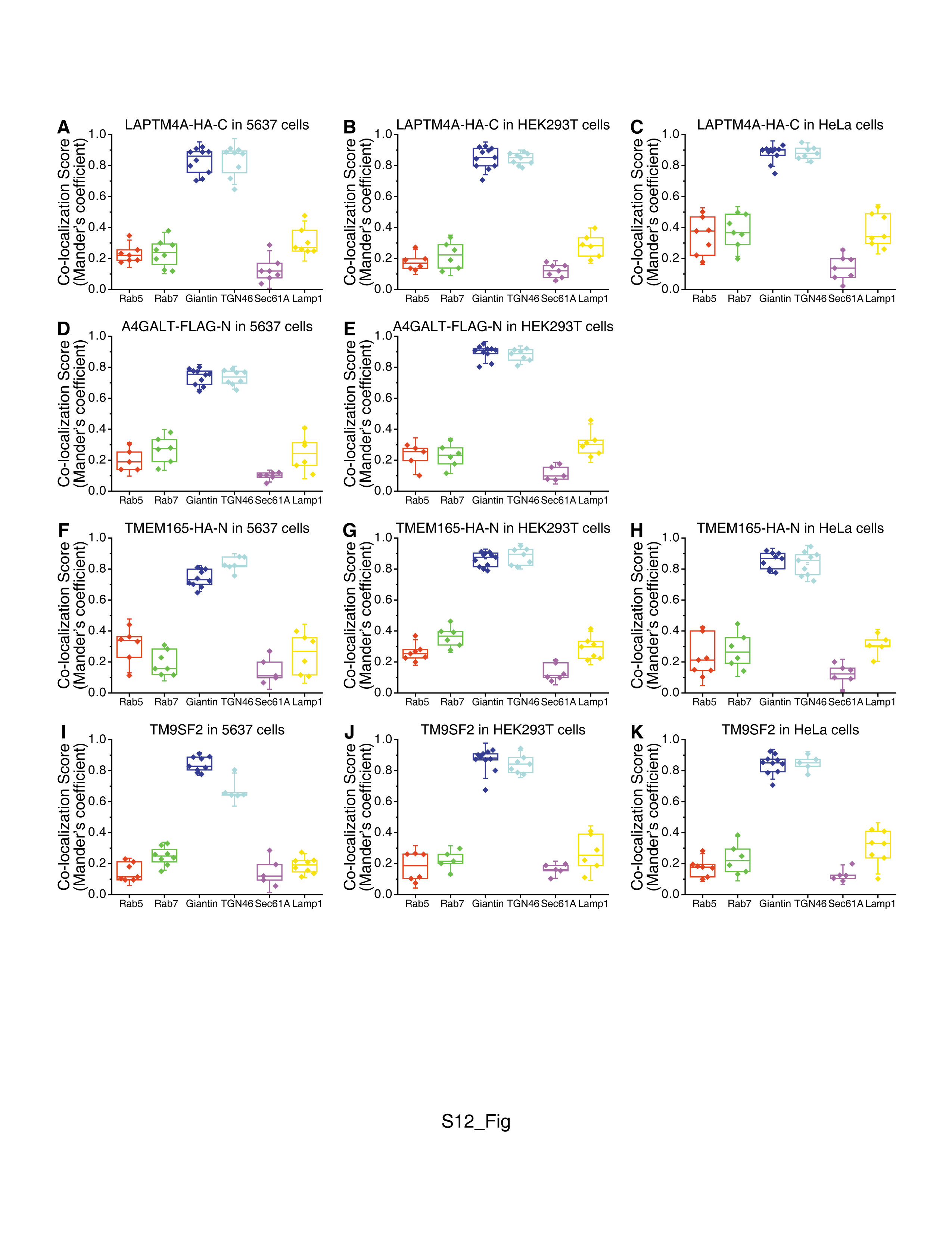

Supplement: S12 Fig — The colocalization of C-terminal HA-tagged LAPTM4A (Fig 3A and S5A and S5B Fig), N-terminal FLAG-tagged A4GALT (S6B and S6C Fig), C-terminal HA-tagged TMEM165 (Fig 5A and S9C and S9D Fig), and endogenous TM9SF2 (Fig 7A and S11B and S11C Fig) with different organelle markers was analyzed using ImageJ. The Mander’s coefficient obtained was utilized as the colocalization score. Six to ten individual cells were analyzed and shown as box charts. Error bars indicate mean ± SD. (TIF) [file pbio.2006951.s012.tif]
