# Supplementary material for: Genome-wide CRISPR screens for Shiga toxins and ricin reveal Golgi proteins critical for glycosylation
Source: PLoS Biol. 2018 Nov 27;16(11):e2006951. doi: 10.1371/journal.pbio.2006951 (PMC6258472; doi:10.1371/journal.pbio.2006951)
Supplement: S1 Table — (DOCX) [file pbio.2006951.s013.docx]

**S1 Table. The IC_50_ of Stx1, Stx2, and Ricin against the indicated cell lines.**

| Cell lines | IC_50_ of Stx1 (ng/mL) | IC_50_ of Stx2 (ng/mL) | IC_50_ of Ricin (ng/mL) |
| --- | --- | --- | --- |
| ACHN*^a^* | 0.613 ± 0.316 | 0.267 ± 0.154 | 11.52 ± 1.44 |
| ACHN-LAPTM4A-KO-Mix | 1113 ± 177 | 86.8 ± 8.8 | N.T. |
| ACHN-A4GALT-KO-Mix | > 10000 | > 10000 | N.T. |
| 5637*^b^* | 0.0278 ± 0.0166 | 0.0074 ± 0.0035 | 0.0549 ± 0.0122 |
| 5637-LAPTM4A-KO-Mix | 901 ± 165 | 246 ± 88 | N.T. |
| 5637-LAPTM4A-KO-II-Mix | 6755 ± 985 | 5321 ± 912 | N.T. |
| 5637-A4GALT-KO-Mix | 6954 ± 872 | 5891 ± 886 | N.T. |
| 5637-SLC35A2-KO-Mix | 7104 ± 475 | 4732 ± 442 | N.T. |
| 5637-UGCG-KO-Mix | 37981 ± 3044 | 27070 ± 2123 | N.T. |
| 5637-B4GALT5-KO-Mix | 8811 ± 1418 | 7038 ± 1904 | N.T. |
| 5637-TMEM165-KO-Mix | 8.49 ± 1.15 | 6.82 ± 3.24 | N.T. |
| 5637-TM9SF2-KO-Mix | 634 ± 55 | 515 ± 166 | N.T. |
| 5637-LAPTM4B-KO-Mix | 0.0121 ± 0.0043 | 0.0076 ± 0.0032 | N.T. |
| HeLa*^c^* | 2155± 159 | 579 ± 101 | 0.0078 ± 0.0009 |
| HeLa-MGAT2-KO-Mix | N.T. | N.T. | 0.290 ± 0.021 |
| HeLa-SLC35C1-KO-Mix | N.T. | N.T. | 0.104 ± 0.014 |
| HeLa-GOSR1-KO-Mix | N.T. | N.T. | 1.59 ± 0.34 |
| HeLa-ERP44-KO-Mix | N.T. | N.T. | 0.293 ± 0.044 |
| HeLa-JTB-KO-Mix | N.T. | N.T. | 0.208 ± 0.011 |
| HeLa-TAPT1-KO-Mix | N.T. | N.T. | 0.0978 ± 0.0141 |
| HeLa-NBAS-KO-Mix | N.T. | N.T. | 0.376 ± 0.035 |
| HT29*^d^* | > 10000 | 9746 ± 739 | N.T. |
| Caco-2*^e^* | > 10000 | > 10000 | N.T. |
| U2OS*^f^* | > 10000 | > 10000 | N.T. |
| A498*^g^* | > 10000 | > 10000 | N.T. |
| A549*^h^* | > 10000 | > 10000 | 4.21 ± 0.87 |
| T24*^i^* | > 10000 | > 10000 | N.T. |
| RT4*^j^* | > 10000 | > 10000 | N.T. |

N.T.: Not tested

*^a^* renal cell adenocarcinoma, kidney (ATCC#: CRL-1611); *^b^* grade II carcinoma, urinary bladder (ATCC#: HTB-9); *^c^* adenocarcinoma, cervix (ATCC#: CCL-2); *^d^* colorectal adenocarcinoma, colon (ATCC#: HTB-38); *^e^* colorectal adenocarcinoma, colon (ATCC#: HTB-37); *^f^* osteosarcoma, bone (ATCC#: HTB-96); *^g^* carcinoma, kidney (ATCC#: HTB-44); *^h^* carcinoma, lung (ATCC#: CRM-CCL-185); *^I^* transitional cell carcinoma, urinary bladder (ATCC#: HTB-4); *^j^* transitional cell papilloma, urinary bladder (ATCC#: HTB-2).
