# Supplementary material for: Genome-wide CRISPR screens for Shiga toxins and ricin reveal Golgi proteins critical for glycosylation
Source: PLoS Biol. 2018 Nov 27;16(11):e2006951. doi: 10.1371/journal.pbio.2006951 (PMC6258472; doi:10.1371/journal.pbio.2006951)
Supplement: S2 Table — (DOCX) [file pbio.2006951.s014.docx]

**S2 Table. The sgRNA sequence for particular genes used to generate KO cell lines.**

| Gene | sgRNA sequence |
| --- | --- |
| LAPTM4A | TCATACTGAATGTTGACAGC |
| LAPTM4A-II | TTCTCCAGTATCAAGTGGGT |
| A4GALT | CAGTCCCGCTACGTCCTCAA |
| SLC35A2 | CCTAGGTAACGTGAAGCACC |
| UGCG | CCGATTACACCTCAACAAGA |
| B4GALT5 | GATCGCAACTATTATGGATG |
| TMEM165 | TTACTGCCAATCCCGTGCAC |
| TM9SF2 | AAGCGCCCATCTGAAAATCT |
| LAPTM4B | CTTGAAAAGTTATACTGATC |
| MGAT2 | GGTGCTAATCCTGACGCTCG |
| SLC35C1 | GCTAGCCAGCACGCCGAAGA |
| GOSR1 | CCCTGATGCATACATTACAG |
| ERP44 | CAGATGCTCCCTTCTGCTCC |
| JTB | TGACAAGACAAGCGAAGATC |
| TAPT1 | GATAGTGTGTCTAAGAAACA |
| NBAS | AACATGGCGGCCCCCGAGTC |
