# Supplementary material for: Genome-wide CRISPR screens for Shiga toxins and ricin reveal Golgi proteins critical for glycosylation
Source: PLoS Biol. 2018 Nov 27;16(11):e2006951. doi: 10.1371/journal.pbio.2006951 (PMC6258472; doi:10.1371/journal.pbio.2006951)
Supplement: S3 Table — (DOCX) [file pbio.2006951.s015.docx]

**S3 Table. Genotypes of KO single clones and their relative sensitives to toxins (compared to WT cells).**

| Single clone | Gentype | Relative sensitivity of Stx1 (fold) | Relative sensitivity of Stx2 (fold) | Relative sensitivity of Ricin (fold) | Relative sensitivity of A-Dtx (fold) | Relative sensitivity of Ctx (fold) |
| --- | --- | --- | --- | --- | --- | --- |
| LA-Mut-9 | Shift (-14 bp)  Shift (-4 bp)  Delete (-9 bp) | 61.7 ± 21.8 | 8.39 ± 4.17 | 0.95 ± 0.28 | 0.79 ± 0.51 | 0.99 ± 0.21 |
| LA-KO-10 | Shift (+1 bp) Shift (-2 bp) Shift (-1 bp) | > 10000 | > 10000 | 1.02 ± 0.15 | 0.66 ± 0.17 | 0.78 ± 0.04 |
| LA-KO-12 | Shift (-28 bp) Shift (-14 bp) Shift (-26 bp) | > 10000 | > 10000 | 1.11 ± 0.39 | 0.91 ± 0.24 | 0.67 ± 0.04 |
| TM-Mut-1 | Shift (-5 bp) Insert (+6 bp) | 39.8 ± 9.4 | 32.4 ± 14.5 | 1.40 ± 0.44 | 1.70 ± 1.20 | 2.67 ± 0.21 |
| TM-KO-3 | Shift (-11 bp) Shift (-10 bp) | 22.2 ± 9.2 | 81.5 ± 21.1 | 1.57 ± 0.68 | 0.98 ± 0.12 | 4.70 ± 1.21 |
| SF2-WT-5 | WT  Shift (+1 bp) | 11.6 ± 3.8 | 4.52 ± 0.85 | 1.08 ± 0.22 | 0.98 ± 0.67 | 1.09 ± 0.35 |
| SF2-KO-8 | Shift (+1 bp) Shift (-7 bp) | > 10000 | > 10000 | 2.32 ± 1.25 | 0.11 ± 0.04 | 5.24 ± 1.55 |
| SF2-KO-9 | Shift (-4 bp) Shift (-28 bp) Shift (+1 bp) | > 10000 | > 10000 | 2.98 ± 1.06 | 2.43 ± 0.46 | 5.21 ± 1.30 |
